# Restructuring breeding programs 2: Assortative mating for improved commercial genetic gain when using optimum contribution selection and diversity introduction

**DOI:** 10.1186/s12711-026-01049-6

**Published:** 2026-05-27

**Authors:** Tobias A. M. Niehoff, Jan ten Napel, Torsten Pook, Mario P. L. Calus

**Affiliations:** Animal Breeding and Genomics, Wageningen University & Research, Droevendaalsesteeg 1, P.O. Box 338, Wageningen, 6700AH The Netherlands

## Abstract

**Background:**

Commercial breeding programs have two goals: genetic gain in the breeding population and delivering competitive genetic products, like semen, to production farms. Breeding programs must also manage genetic diversity, which reduces short-term genetic gain. Our study aimed to investigate the use of assortative mating to increase the genetic variance of the next generation. The resulting higher genetic variance is expected to increase the genetic level of the best animals of which semen can be sold to producers. We are here particularly interested in applying this strategy in breeding programs that invest in diversity introduction by bringing lost or lowly related alleles from lower-performing sources into the elite nucleus breeding population. We test two assortative mating strategies. The first, ‘maximum assortative mating’, aimed to increase the genetic variance as much as possible by mating the best male with the best female, second best with second best and so on. The second, ‘tuned assortative mating’, aimed to increase the genetic variance as much as needed to make the same gain as a hypothetical competitor that uses random mating. The motivation for the tuned strategy was to limit anticipated negative effects of assortative mating on genomic prediction accuracies.

**Results:**

The ‘tuned’ strategy was outperformed by the ‘maximum’ strategy, and no negative effects on prediction accuracy or biases due to the mating strategy were found. With truncation selection, i.e., no control of the inbreeding rate, assortative mating led to a faster decrease of genetic diversity over time compared to random mating. Assortative mating combined with optimum contribution selection (OCS) showed a comparable inbreeding rate to random mating, and led to higher variance of breeding values. While achieving the same genetic gain of the population average, OCS with assortative mating thus led to a higher genetic level of the elite boars from which semen was marketed.

**Conclusions:**

Assortative mating improves the genetic level of outputs of breeding programs but this effect was not large enough to overcome the lower competitiveness associated with diversity introduction. To limit the accelerated loss of diversity, assortative mating should only be used when selection considers diversity, as in OCS.

**Supplementary Information:**

The online version contains supplementary material available at 10.1186/s12711-026-01049-6.

## Background

Commercial nucleus breeding programs have two primary goals: genetic improvement of the nucleus breeding population and delivering competitive genetic products, like semen, to production farms. At the same time, breeding programs must manage genetic diversity, which reduces the selection pressure that can be applied to improve the population. The genetic level of outputs of a breeding program represents the immediate return on investment of selection. Improving the diversity of a population represents investment for which returns can only be expected in the future when the diversity proves to be valuable.

Although breeders typically understand that diversity is important [1], it can be difficult to pinpoint for which purpose (“what are important traits in the future?”) and the time horizon (“when do new traits become important?”) for which variation is needed. The only option breeders have is to manage overall, trait unspecific, diversity, in hopes that overall diversity is overlapping with diversity of traits that will be important in the future, and doing this without jeopardizing the genetic progress of the population.

In [2], we tested the design of multi-layer breeding programs for the integration of external diversity that is missing from the elite nucleus breeding population. In essence, a fraction of the females that would normally be selected to produce the next generation of elite animals is instead used for backcrossing to descendants of diversity donor x elite matings. Those matings are used to upgrade the genetic level of descendants of diversity donors for the traits under selection. As in [2], diversity donors are males that were selected as sires at some point in the past and of which semen is cryo-stored. As a consequence of reducing the number of elite x elite matings, the size of the elite breeding population is reduced, as well as the number of elite selection candidates in the next generation. Assuming that breeding programs want to sell (semen of) a fixed number of animals, e.g. boars for artificial insemination (AI) stations to be used for dissemination of genetics to production farms, this means that the selection intensity for those boars will be lower and the short-term commercial genetic gain (improvement of output of breeding program) will be lower.

**Fig. 1:**
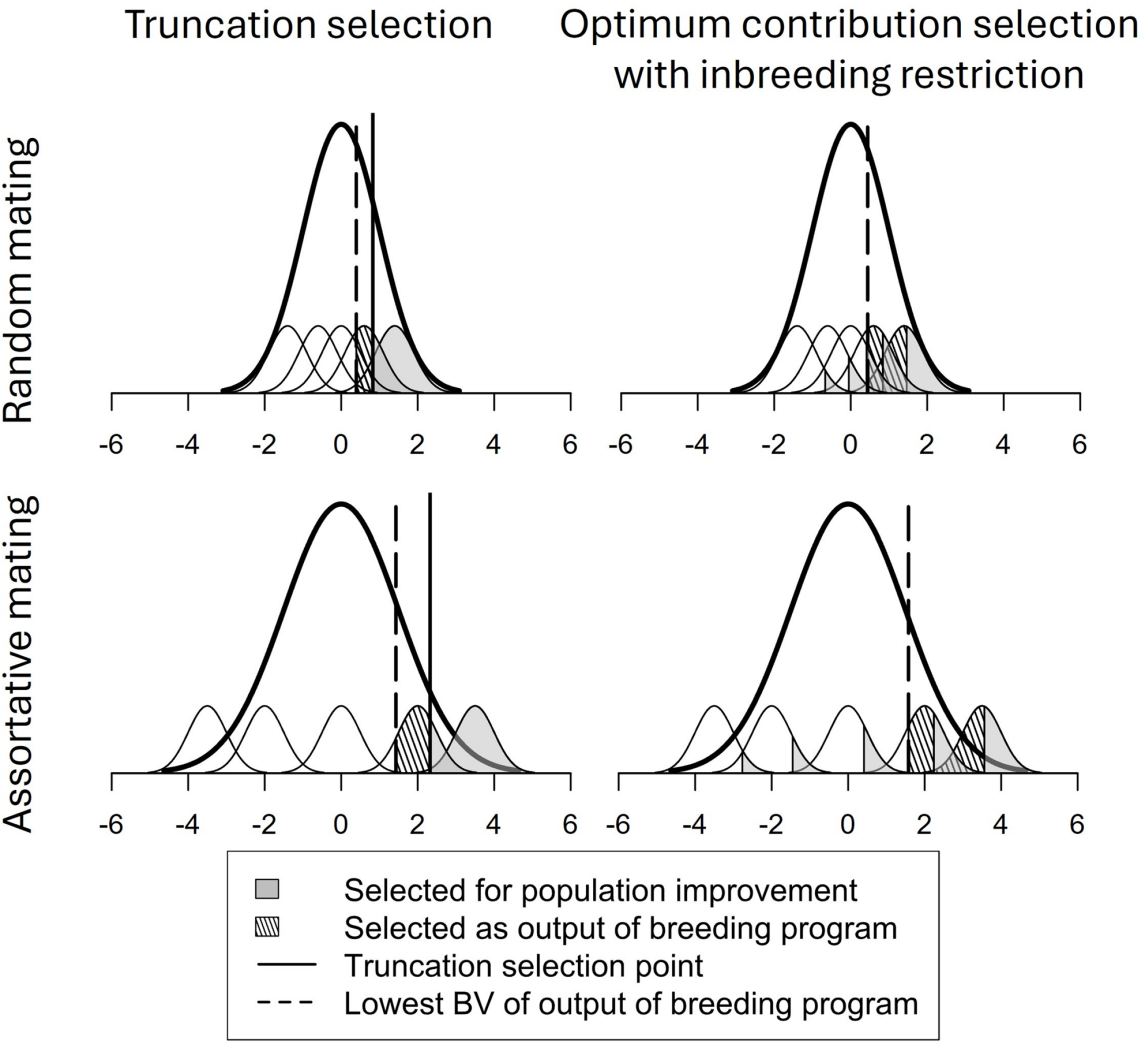
Visualization of contributions of unrelated non-inbred families to elite parents (grey) and AI animals (shaded) when using truncation selection and Optimum contribution selection after the population was created with random and assortative mating. The figure shows the principle and is not to scale.With truncation selection and random mating, animals from 3 families are selected for population improvement whereas only 2 families contribute with assortative mating of parents. When selecting with OCS with a restriction of the inbreeding rate (in this example using pedigree kinships), the proportions of selected animals per family are more equalized among families and identical among mating strategies. The genetic level of AI animals is higher under assortative mating.

The idea of this study is to use assortative mating, i.e., mating the best sire with the best dam, the second best with the second best, and so on, to artificially increase the variance of breeding values of selection candidates in the next generation, which in turn would mean that the genetic level of the top animals, i.e., AI boars, will be greater. This is visualized in Fig. 1 on the left side. “AI boars” in this study are boars that are not selected for population improvement but whose semen is sold to production farms to produce finisher pigs or multipliers. Their average (estimated) breeding values represent the commercial genetic level. Here, we test the effects of assortative mating in combination with the simulated upcycling breeding program that introduces genetic diversity into a breeding nucleus, as described in [2]. In this context, assortative mating can be seen as a means to increase genetic variance and, thus, genetic gain in the short-term, whereas diversity introduction is a long-term investment in genetic variance and thus in long-term genetic gain. The idea to combine both is to obtain the long-term benefits of diversity introduction without the disadvantages of reduced genetic gain in the short-term without the use of assortative mating.

The increase in genetic variance achieved by assortative mating is more specifically caused by an increase in the variance of family means in the next generation. Therefore, applying truncation selection based on estimated breeding values (EBVs) to a population that has been generated by assortative mating results in a greater imbalance of contributions of families to the next generation, which in turn increases the inbreeding rate [3, p. 67–69], because the rate of inbreeding is proportional to the sum of squared founder contributions ($\Delta F \propto \sum {contr^{2} }$) [4]. In other words, under assortative mating, many animals are selected from a few families, while less or none are selected from other families, and this imbalance is greater than if the families were created with random mating (visualized in Fig. 1). This results in selecting more related animals, which increases the inbreeding rate and the loss of genetic variation. Previous studies that did not consider the effects of inbreeding on genetic variance did not find adverse effects of assortative mating (e.g. [5–7]), but several more recent simulation studies have shown that successive generations of assortative mating with truncation selection increases the rate of inbreeding [8–11]. We hypothesize that the elevated inbreeding rate can be controlled by using optimum contribution selection (OCS) [12], such that the positive effect of assortative mating is added without negative side effects. Results of simulation studies based on the infinitesimal model in forest tree breeding using mass selection and comparable forms of inbreeding control support this hypothesis [13, 14]. OCS finds a balance of between-family selection and within-family selection and is expected to result in the same targeted rate of inbreeding, regardless of the spread of family means. Note that, under random mating, the rate of inbreeding is the identical to the rate of increase in population-average kinship. Controlling the latter, which is the purpose of OCS, is the actual aim of maintaining diversity. For brevity and because of their identity under random mating, we use the term inbreeding rate in this study.

The consequences of assortative mating on the accuracy of genomic prediction have not been studied extensively before. Ansari et al. [15] showed that assortative mating of parents increases the accuracy of EBVs in the next generation. This is likely caused by greater variance of family means, which increases the variance of true breeding values (TBV), and thereby the prediction accuracy, i.e. the correlation between TBV and EBV. Ansari et al. [15] only simulated a single generation and the genomic prediction training set was not comprised of animals that are themselves products of assortative mating. We hypothesize that training a genomic prediction model with data of animals that have been produced by assortative mating might negatively impact the prediction accuracy, i.e., result in larger prediction errors, or lower correlation between EBV and TBV for a given genetic variance. This is because assortative mating will lead to a greater imbalance of the distribution of beneficial alleles across families, i.e., many beneficial alleles will be found together in some families and many unfavorable alleles in families at the lower end of the distribution. Consequently, this non-random association of alleles with family means, or disequilibrium, may make it more difficult for a model to infer QTL effects correctly. This non-random association may not be harmful when predictions are solely based on own phenotypes, as suggested by observed continuous gain in previous studies on assortative mating, e.g. [13, 14], but it might harm the accuracy of genomic predictions, specifically with little or none own or offspring phenotypic information.

In an attempt to limit potential unwanted side-effects of non-random mating, we tested a strategy called ‘tuned assortative mating’ in which mate allocation was conducted that aims to achieve a correlation between the TBV of males and their allocated females that matches a predetermined value (in this study, always between 0 and 1). That value was set such that the variance of breeding values was large enough for the commercial genetic level (here the average TBV or EBV of AI boars) to be the same as the average commercial genetic level of a (hypothetical) competitor breeding program. The hypothetical competitor breeding program was initially equivalent to the own breeding program, but did not invest in diversity introduction and, therefore, spent all resources on advancing the elite. To our knowledge, such a tuned strategy has not been evaluated before. Studies typically conduct mate allocation such that the correlation between TBVs of males and females is as close to 1 as possible, which we call ‘maximum assortative mating’. Some studies include a ‘disassortative’ mating as a control, which involve mating genetically good animals to bad animals, and we also included this strategy. Assortative and disassortative mating are sometimes also referred to as ‘positive’ and ‘negative’ assortative mating [8, 10].

Against this background, the aim of this study was to examine, in the context of genomic selection, the effects of assortative mating on genetic gain of the nucleus population and merit of selected AI sires, diversity, and genomic prediction accuracies, using stochastic simulation. In the first part of this study, we compared the outcomes of truncation selection and OCS under different mating schemes to demonstrate the described effects on genetic gain and diversity. In the second part, we tested assortative mating in a multi-layer breeding program with diversity introduction.

## Methods

### Derivation of the required correlation between breeding values of sires and dams

For our “tuned assortative mating strategy”, the goal was that the animals selected as AI boars in the next generation have the same genetic level as those of a competitor breeding program that uses a random mating strategy. Note that we use the competitor as a hypothetical case which resembles switching the own breeding program from a multi-layer program to a standard program without diversity introduction in the next generation. The same number of AI boars should be selected in the own and in the competitor breeding program. Since the elite population size of the own breeding program is reduced due to diverting resources towards diversity introduction, this would lower the selection intensity. To match the genetic level of AI boars of the competitor, the genetic variance needs to be increased through assortative mating. The parameter to tune, therefore, is the covariance of EBVs of males and their mates, as this increases the variance of family means, or the variance of parent average breeding values of animals in the next generation Eq. (1). We need to find out by how much the variance in the own breeding program needs to be increased to achieve the same level as the competitor (Eqs. (2) and (3)). The control scenario in this study is a breeding program that never invested in diversity introduction. In the first generation of the simulation, the own breeding program and that of the hypothetical competitor, the control, are equivalent.

Equation (1) is taken from Eq. 16.21b of Walsh and Lynch [16]. The term $\:{{\upsigma\:}}_{{\mathrm{T}\mathrm{B}\mathrm{V}}_{\mathrm{t}+1}}^{2}$ describes the variance of TBVs in the next generation (t + 1):

1 $$ \begin{aligned} \upsigma _{{{\mathrm{TBV}}_{{{\mathrm{t}} + 1}} }}^{2} = & \frac{{\upsigma _{{{\mathrm{TBV}}_{{{\mathrm{sires}}_{{\mathrm{t}}} }} }}^{2} }}{4} + \frac{{\upsigma _{{{\mathrm{TBV}}_{{{\mathrm{dams}}_{{\mathrm{t}}} }} }}^{2} }}{4} + \frac{{{\mathrm{COV}}\left( {{\mathrm{TBV}}_{{{\mathrm{sires}}_{{\mathrm{t}}} }} ,{\mathrm{TBV}}_{{{\mathrm{dams}}_{{\mathrm{t}}} }} } \right)}}{2} \\ & + \upsigma _{{{\mathrm{gamMS}}_{{{\mathrm{sires}}_{{\mathrm{t}}} }} }}^{2} + \upsigma _{{{\mathrm{gamMS}}_{{{\mathrm{dams}}_{{\mathrm{t}}} }} }}^{2} \\ \end{aligned} $$

where $$ \frac{{\sigma _{{{\mathrm{TBV}}_{{{\mathrm{sires}}_{{\mathrm{t}}} }} }}^{2} }}{4} + \frac{{\sigma _{{{\mathrm{TBV}}_{{{\mathrm{dams}}_{{\mathrm{t}}} }} }}^{2} }}{4}$$ +$$ \frac{{{\mathrm{COV}}\left( {{\mathrm{TBV}}_{{{\mathrm{sires}}_{{\mathrm{t}}} }} ,\;{\mathrm{TBV}}_{{{\mathrm{dams}}_{{\mathrm{t}}} }} } \right)}}{2} $$

 is the variance of parent average TBVs in the next generation, also called between-family variance, comprised of $\upsigma_{{TBV}_{{sires}_{t}}}^{2}$ and $\upsigma_{{TBV}_{{dams}_{t}}}^{2}$ as the variance of TBVs of selected males and females, respectively, and $\:\mathrm{C}\mathrm{O}\mathrm{V}({\mathrm{T}\mathrm{B}\mathrm{V}}_{{\mathrm{s}\mathrm{i}\mathrm{r}\mathrm{e}\mathrm{s}}_{\mathrm{t}}},{\mathrm{T}\mathrm{B}\mathrm{V}}_{{\mathrm{d}\mathrm{a}\mathrm{m}\mathrm{s}}_{\mathrm{t}}})$ as the covariance between TBVs of sires and their mates; $\upsigma_{{\mathrm{g}\mathrm{a}\mathrm{m}\mathrm{M}\mathrm{S}}_{{\mathrm{s}\mathrm{i}\mathrm{r}\mathrm{e}\mathrm{s}}_{\mathrm{t}}}}^{2}$ and $\upsigma_{{\mathrm{g}\mathrm{a}\mathrm{m}\mathrm{M}\mathrm{S}}_{{\mathrm{d}\mathrm{a}\mathrm{m}\mathrm{s}}_{\mathrm{t}}}}^{2}$ are the variances of Mendelian sampling terms generated by selected sires and dams, respectively, also called gametic Mendelian sampling variances. The sum of the two latter terms is the within-family variance.

Based on the difference of average TBV of AI boars of the competitor ($\overline{{{\mathrm{TBV}}}} _{{{\mathrm{AI}}\:{\mathrm{competitor}}}}$), which we can assume to be the same as their average EBV since the regression of TBV on EBV is expected to be 1, and the average EBV of the selected animals in the own program ($\:{\mathrm{P}\mathrm{A}}_{\mathrm{E}\mathrm{B}\mathrm{V}\:\mathrm{o}\mathrm{w}\mathrm{n}}$) (which is expected to be the same as the average TBV) as well as the to-be-applied intensity for selecting AI boars ($\:{\mathrm{i}}_{\mathrm{o}\mathrm{w}\mathrm{n}}$), the required standard deviation of EBVs in the own program ($\upsigma_{{\mathrm{E}\mathrm{B}\mathrm{V}}_{\mathrm{o}\mathrm{w}\mathrm{n}\:\mathrm{r}\mathrm{e}\mathrm{q}\mathrm{u}\mathrm{i}\mathrm{r}\mathrm{e}\mathrm{d}\:\mathrm{t}+1}}$) can be worked out with Eq. 2:

2 $$\upsigma _{{{\mathrm{EBV}}_{{{\mathrm{own}}{\kern 1pt} {\mathrm{required}}{\kern 1pt} {\mathrm{t}} + 1}} }} = \frac{{\left( {\overline{{{\mathrm{TBV}}}} _{{{\mathrm{AI}}\:{\mathrm{competitor}}}} - {\mathrm{PA}}_{{{\mathrm{EBV}}\:{\mathrm{own}}}} } \right)}}{{{\mathrm{i}}_{{{\mathrm{own}}}} }} $$

By assuming that the selection accuracy of the candidates that may be selected as AI boars now (${\mathrm{r}}_{{{\mathrm{TBV,EBV}}_{{{\mathrm{cand}}}} }}^{2}$) is the same as the accuracy in the next generation, the required variance of TBV in the next generation can be worked out as:

3 $$\upsigma _{{{\mathrm{TBV}}_{{{\mathrm{own}}{\kern 1pt} {\mathrm{required}}{\kern 1pt} {\mathrm{t}} + 1}} }}^{2} = \frac{{\upsigma _{{{\mathrm{EBV}}_{{{\mathrm{own}}{\kern 1pt} {\mathrm{required}}{\kern 1pt} {\mathrm{t}} + 1}} }}^{2} }}{{{\mathrm{r}}_{{{\mathrm{TBV}},{\mathrm{EBV}}_{{{\mathrm{cand}}}} }}^{2} }} $$

The required covariance (Eq. (4)) between TBVs of sires and dams ($\mathrm{r}\mathrm{e}\mathrm{q}\mathrm{u}\mathrm{i}\mathrm{r}\mathrm{e}\mathrm{d}\:\mathrm{C}\mathrm{O}\mathrm{V}\left({\mathrm{T}\mathrm{B}\mathrm{V}}_{{\mathrm{s}\mathrm{i}\mathrm{r}\mathrm{e}}_{\mathrm{t}}},{\mathrm{T}\mathrm{B}\mathrm{V}}_{{\mathrm{d}\mathrm{a}\mathrm{m}}_{\mathrm{t}}}\right)$) can be calculated based on the required variance of TBVs from Eq. (3) and the variance of TBVs that would be observed if mating was random ($\upsigma _{{{\mathrm{TBV}}_{{{\mathrm{own}}{\kern 1pt} {\mathrm{rand}}{\kern 1pt} {\mathrm{t}} + 1}} }}^{2}$). The latter can be obtained by inserting all terms in Eq. (1) and assuming $\mathrm{C}\mathrm{O}\mathrm{V}({\mathrm{T}\mathrm{B}\mathrm{V}}_{{\mathrm{s}\mathrm{i}\mathrm{r}\mathrm{e}\mathrm{s}}_{\mathrm{t}}},{\mathrm{T}\mathrm{B}\mathrm{V}}_{{\mathrm{d}\mathrm{a}\mathrm{m}\mathrm{s}}_{\mathrm{t}}})$ to be 0 for random mating:

4 $$\begin{gathered} {\mathrm{required}}\:{\mathrm{COV}}\left( {{\mathrm{TBV}}_{{{\mathrm{sire}}_{{\mathrm{t}}} }} ,{\mathrm{TBV}}_{{{\mathrm{dam}}_{{\mathrm{t}}} }} } \right) \hfill \\ = \left( {\upsigma _{{{\mathrm{TBV}}_{{{\mathrm{own}}\;{\mathrm{required}}\;{\mathrm{t}} + 1}} }}^{2} - \upsigma _{{{\mathrm{TBV}}_{{{\mathrm{own}}\;{\mathrm{rand}}\;{\mathrm{t}} + 1}} }}^{2} } \right){\text{}}2 \hfill \\ \end{gathered} $$

This required covariance is then converted into the required correlation between TBVs of sires and their mates (Eq. (5)). The EBVs and estimated prediction error variances (PEV), which we here derive from the model-based individual reliability, can then be used to derive the correlation between EBVs and TBVs within the group of selected sires (${\mathrm{r}}_{{{\mathrm{EBV}},{\mathrm{TBV}}_{{{\mathrm{sires}}}} }}$) and dams (${\mathrm{r}}_{{\mathrm{E}\mathrm{B}\mathrm{V},\mathrm{T}\mathrm{B}\mathrm{V}}_{\mathrm{d}\mathrm{a}\mathrm{m}\mathrm{s}}}$) (Eq. (6)).

5 $$ {\mathrm{required}}\:{\mathrm{COR}}\left( {{\mathrm{TBV}}_{{{\mathrm{sire}}}} ,{\mathrm{TBV}}_{{{\mathrm{dam}}}} } \right) = \frac{{{\mathrm{required}}\:{\mathrm{COV}}\left( {{\mathrm{TBV}}_{{{\mathrm{sire}}_{{\mathrm{t}}} }} ,{\mathrm{TBV}}_{{{\mathrm{dam}}_{{\mathrm{t}}} }} } \right)}}{{\sqrt {\upsigma _{{{\mathrm{TBV}}\:{\mathrm{sires}}}}^{2} {\mathrm{*}}\upsigma _{{{\mathrm{TBV}}\:{\mathrm{dams}}}}^{2} } }} $$

6 $${\mathrm{r}}_{{{\mathrm{EBV}},{\mathrm{TBV}}_{{{\mathrm{sires}}}} }} = \sqrt {1 - \frac{{\overline{{{\mathrm{PEV}}}} _{{{\mathrm{sires}}}} }}{{\upsigma _{{{\mathrm{EBV}}\:{\mathrm{sires}}}}^{2} + {\mathrm{PEV}}_{{{\mathrm{sires}}}} }}} ({\mathrm{same}}\;{\mathrm{for}}\;{\mathrm{dams}})$$

The correlation between the EBVs required to achieve the required correlation of TBVs can then be worked out based on these correlations of EBVs with TBVs as:

7 $${\mathrm{required}}\:{\mathrm{COR}}\left( {{\mathrm{EBV}}_{{{\mathrm{sire}}}} ,{\mathrm{EBV}}_{{{\mathrm{dam}}}} } \right) = \frac{{{\mathrm{required}}\:{\mathrm{COR}}\left( {{\mathrm{TBV}}_{{{\mathrm{sire}}_{{\mathrm{t}}} }} ,{\mathrm{TBV}}_{{{\mathrm{dam}}_{{\mathrm{t}}} }} } \right)}}{{{\mathrm{r}}_{{{\mathrm{EBV}},{\mathrm{TBV}}_{{{\mathrm{sires}}}} }} {\mathrm{*r}}_{{{\mathrm{EBV}},{\mathrm{TBV}}_{{{\mathrm{dams}}}} }} }} $$

It should be noted that the correlation between sire and dam TBVs cannot be higher than the product of their EBV accuracies (Eq. (7)). This means that assortative mating requires both sexes to have accurate EBVs. All the steps we took in the calculations are explained in the appendix (“Detailed derivation of required correlation between breeding values of sires and dams”).

## Mate allocation strategy

To achieve the required correlation between EBVs of sires and their mates, a mating plan is needed. The method that we developed and used for mate allocation is described in detail in the appendix (“Explanation of the mate allocation algorithm”). In brief, for each mating to perform, we sampled values from a bivariate normal distribution whose correlation matched the required correlation of EBVs between mating pairs. Subsequently, the order of values in the two univariate normal distributions was derived to determine the pair-wise order of individual samplings. For example, when the highest realized value for the first normal distribution is observed with the third highest realized value in the second normal distribution, a mating between the best sire and third-best dam is performed.

## Simulation

Our study consists of two simulations. In the first simulation, we compared strategies using random mating, assortative mating, and disassortative mating in combination with truncation selection on EBVs or OCS. Disassortative mating was included as another control scenario. No upcycling scheme with diversity introduction was simulated in this first simulation. Since this makes the tuned assortative mating strategy not meaningful, that strategy was not included. Matings were constructed after the contributions of males were determined with OCS. When males were selected by truncation selection, every male had the same contribution. Females were always selected by truncation selection on EBVs. The purpose of the first simulation was to understand the difference between truncation selection and OCS and the differences between the mating strategies.

In the second simulation, we investigate the effects of tuned assortative mating and maximum assortative mating in breeding programs with diversity introduction. Here, the males were always selected using OCS, while females for the elite were selected based on truncation selection. In this second simulation, the effects on genetic diversity and the accuracy of EBVs were of particular interest.

Both simulations use the same breeding program and parameters as outlined in [2], unless stated otherwise. In brief, a genomic selection swine breeding program was simulated with 2,400 animals per generation generated from 40 sires and 400 dams using the R package MoBPS [17]. All animals were genotyped with a 20k SNP chip and were phenotyped for a trait with heritability 0.3 in the base generation. Data from the current generation plus the previous 5 generations, i.e., 14,400 genotyped and phenotyped animals, were used as the genomic prediction training set. When selection was done with OCS, the number of selected males differed from 40 and contributions of males where optimized to maximize genetic gain under the constraint that the inbreeding rate should not exceed 1%. OCS was implemented with the R package optiSel [18]. Diversity donors were selected based on having the lowest average segment-based kinship to the elite population. This was calculated with the function “segIBD()” of optiSel. Diversity introduction was done by backcrossing in four upcycling layers. To accommodate resources in terms of farrowing pens and dams needed for the layers, the size of the elite population was reduced so that the sum of animals in the layers and elite was the same for all tested scenarios. We used 40 sows (i.e. 10%) for upcycling, instead of the 25% used by Niehoff et al. [2], such that the elite sow number was only reduced from 400 to 360. We assumed that the best 80 boars not selected for population improvement were used to produce semen to be sold to the market. The average genetic level of these “AI boars” represents the commercial genetic level. Twenty replicates were run for every scenario. For a full description, see Niehoff et al. [2].

## Calculation of prediction accuracy and bias

Genomic prediction accuracies are reported as the correlation between EBVs and TBVs for the training population, for elite selection candidates, and for elite sires that were active 10 years ago. Since assortative mating increases the genetic variance and thus the correlation between EBVs and TBVs, we also report the within-family accuracy for the training population and elite candidates. The within-family variance is not affected by the mating strategy, and thus the within-family accuracy allows for a fairer comparison between methods, and tests if the mating strategy affects genomic predictions. The within-family accuracy was calculated as the correlation of EBVs and TBVs per litter and averaged over all litters and can also be understood as the correlation between true and estimated Mendelian sampling terms. In total, we report five different accuracies.

Level bias was calculated as the average difference between true and estimated breeding values in genetic standard deviation units (${\widehat{\upsigma\:}}_{A}$) estimated using rrBLUP [19] based on 3000 random elite animals selected from the three most recent generations. A level bias value of 0 is ideal, while a negative level bias indicates that EBVs overestimate TBVs on average [20]. The vector of EBVs (${\mathbf{ebv}}$) was calculated as

$$ {\mathbf{ebv}} = \hat{\alpha }^{T} ({\mathbf{M}} - 2{\mathbf{P}}) $$

where $\hat{\alpha }$ is the vector of estimated SNP effects and **M** is the genotype matrix, which is centered by subtracting twice the allele frequency observed among all animals included in the evaluation ($2{\mathbf{P}}$) from each individual’s allele count, coded as 0, 1, and 2. Matrices ${\mathbf{M}}$ and ${\mathbf{P}}$ have as many rows as SNPs and as many columns as animals.

The average EBV of the animals in the training set was 0. In the simulation, TBVs are the result of the true QTL effects multiplied with the allele counts and thus not centered to 0. To calculate level bias, the TBVs were centered by the average TBV of the training set in the breeding value estimation ($\overline{{{\mathrm{TBV}}}} _{{\mathrm{BVE}}}$). Level bias was calculated as the difference between the average centered TBV and the average EBV expressed in units of estimated genetic standard deviations:

$$ {\text {Level}\:{\mathrm{bias}}} = \frac{{\frac{{\sum\nolimits_{{{\mathrm{i}} = 1}}^{{\mathrm{n}}} {{\mathrm{TBV}}_{{\mathrm{i}}} } - \overline{{{\mathrm{TBV}}}} _{{{\mathrm{BVE}}}} }}{{\mathrm{n}}} - \frac{{\sum\nolimits_{{{\mathrm{i}} = 1}}^{{\mathrm{n}}} {{\mathrm{EBV}}_{{\mathrm{i}}} } }}{{\mathrm{n}}}}}{{\sqrt {\hat{\upsigma }_{{\mathrm{A}}}^{2} } }} $$

Dispersion bias was estimated as the slope of the regression of TBVs on EBVs, as in Jibrila et al. [20]. A value of 1 means no dispersion bias, while a value lower than 1 means that the variance of EBVs is inflated, i.e., a change of 1 unit on the EBV scale results in less than 1 unit change on the TBV scale.

## Results

### Simulation 1: truncation selection versus Optimum contribution selection

**Fig. 2:**
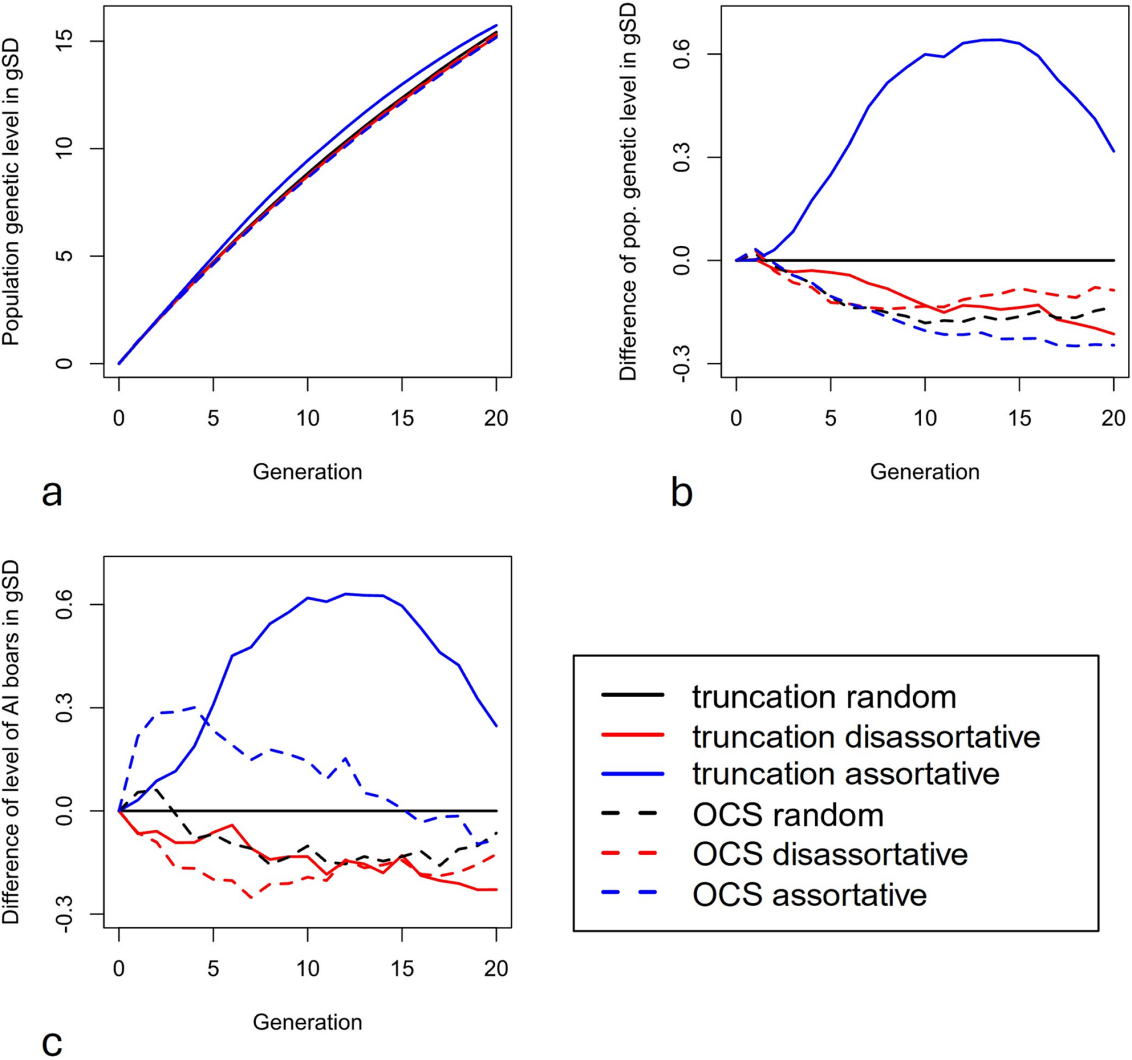
Comparison of genetic gain for truncation selection and optimum contribution selection for different mating strategies. **a** Cumulative genetic gain of the population, **b** difference of population average genetic level relative to that of truncation selection with random mating in genetic standard deviations, and **c** difference of genetic level of AI boars relative to that of truncation selection with random mating in genetic standard deviations. The genetic standard deviation was calculated as the square root of the genic variance in generation 0. The genic standard deviation was approximately 0.92

Assortative mating increased genetic gain (Fig. 2). Truncation selection on GEBVs yielded comparable genetic progress as OCS (Fig. 2a). Compared to random mating, assortative mating resulted in higher genetic levels of AI boars by up to 0.6 and 0.3 genetic standard deviations for truncation selection and OCS, respectively (Fig. 2c). However, assortative mating only resulted in a faster genetic gain, i.e., population average BV change, with truncation selection but not with OCS (Fig. 2b). The standard error of the mean difference to truncation selection with random mating ranged between 0.09 and 0.14 gSD in generation 20. Thus, only assortative mating with truncation selection and assortative mating with OCS were significantly (*p* < 0.05) different from random mating with truncation selection (compare Fig. 2b).

**Fig. 3:**
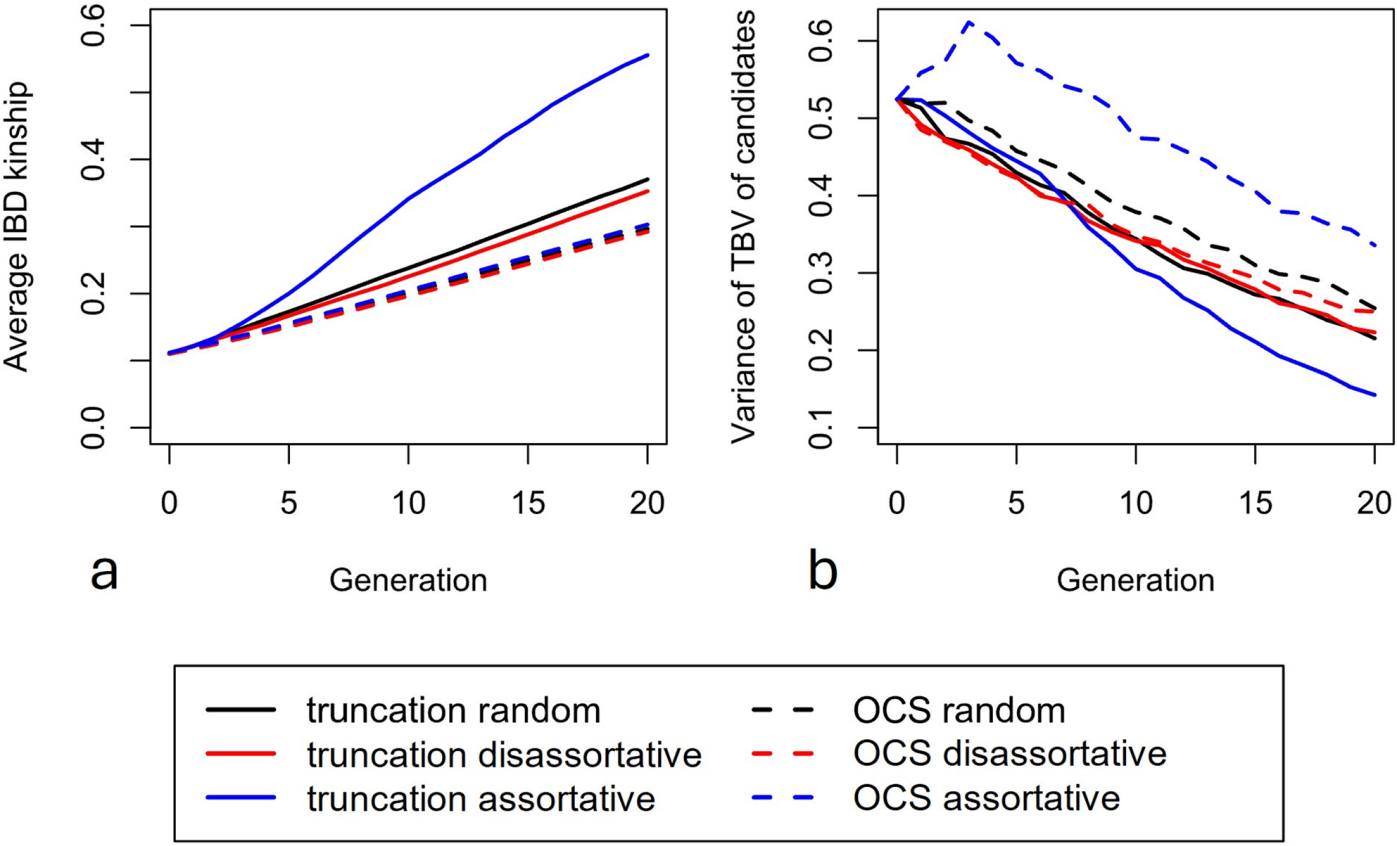
Comparison of diversity for truncation selection and optimum contribution selection for different mating strategies. **a** Average identity-by-descent kinship in the population, and **b** variance of true breeding values among selection candidates

Under truncation selection, assortative mating led to higher inbreeding rates, i.e., increase of average kinship per generation, than random mating (2.22 vs. 1.30% per generation, respectively), while disassortative mating slightly reduced it (1.21%) (Fig. 3a). Populations selected with OCS showed the lowest increase in kinship. Regardless of the mating strategy, OCS kept the inbreeding rate close to the targeted value of 1% (0.93, 0.91, and 0.96% for random, disassortative, and assortative mating, respectively) (Fig. 3a).

The variance of TBV of selection candidates decreased over time (Fig. 3b). The strongest decrease was observed for assortative mating coupled with truncation selection, probably due to the elevated inbreeding rate. However, even with truncation selection, assortative mating did achieve higher genetic variance compared to the other mating strategies but this effect was very small and only observed until generation 6 (Fig. 3b). Coupled with OCS, assortative mating caused considerably higher genetic variance among selection candidates than all other mating scenarios, as expected. Compared to random mating, disassortative mating resulted in slightly lower genetic variances.

## Simulation 2: assortative mating with multi-layer breeding programs

When investing 25% of resources in diversity introduction, as in [2], the required correlation between EBVs of sires and dams in tuned assortative mating to achieve a genetic level of AI boars comparable to that of the competitor Eq. (7) was often greater than 1 but such a correlation is obviously impossible to achieve. Thus, we present results for breeding programs investing only 10% of resources in diversity introduction, as the required correlation between EBVs of sires and dams was always lower than 1.

**Fig. 4:**
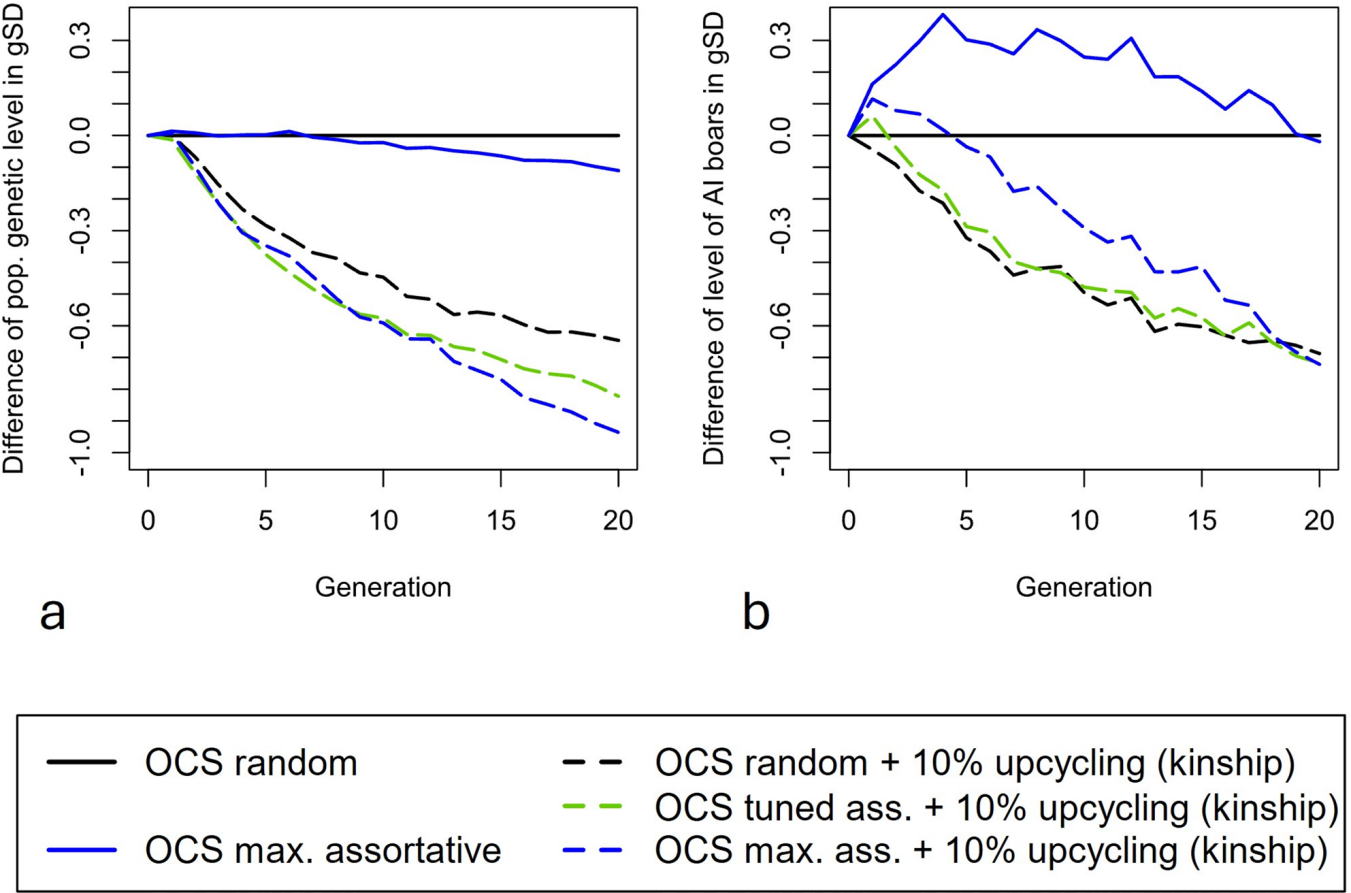
Genetic gain in breeding programs with and without diversity introduction under different mating strategies. Difference of elite population average breeding value compared to OCS with random mating in genetic SD (**a**), and difference of average breeding value of 80 AI boars compared to that of OCS with random mating in genetic SD (**b**). “OCS random” is used as the standard here and its genetic level is centred to 0 in every generation. The genetic standard deviation was calculated as the square root of the genic variance observed in generation 0, which was approximately 0.85. “max. assortative” refers to assortative mating with the highest possible correlation between EBVs of sires and dams. “tuned ass.” refers to assortative mating where matings are constructed in such a way that the correlation between EBVs of sires and dams matches the required correlation

Both tuned and maximum assortative mating only had a small beneficial effect on commercial genetic gain in the short term (Fig. 4).

**Fig. 5:**
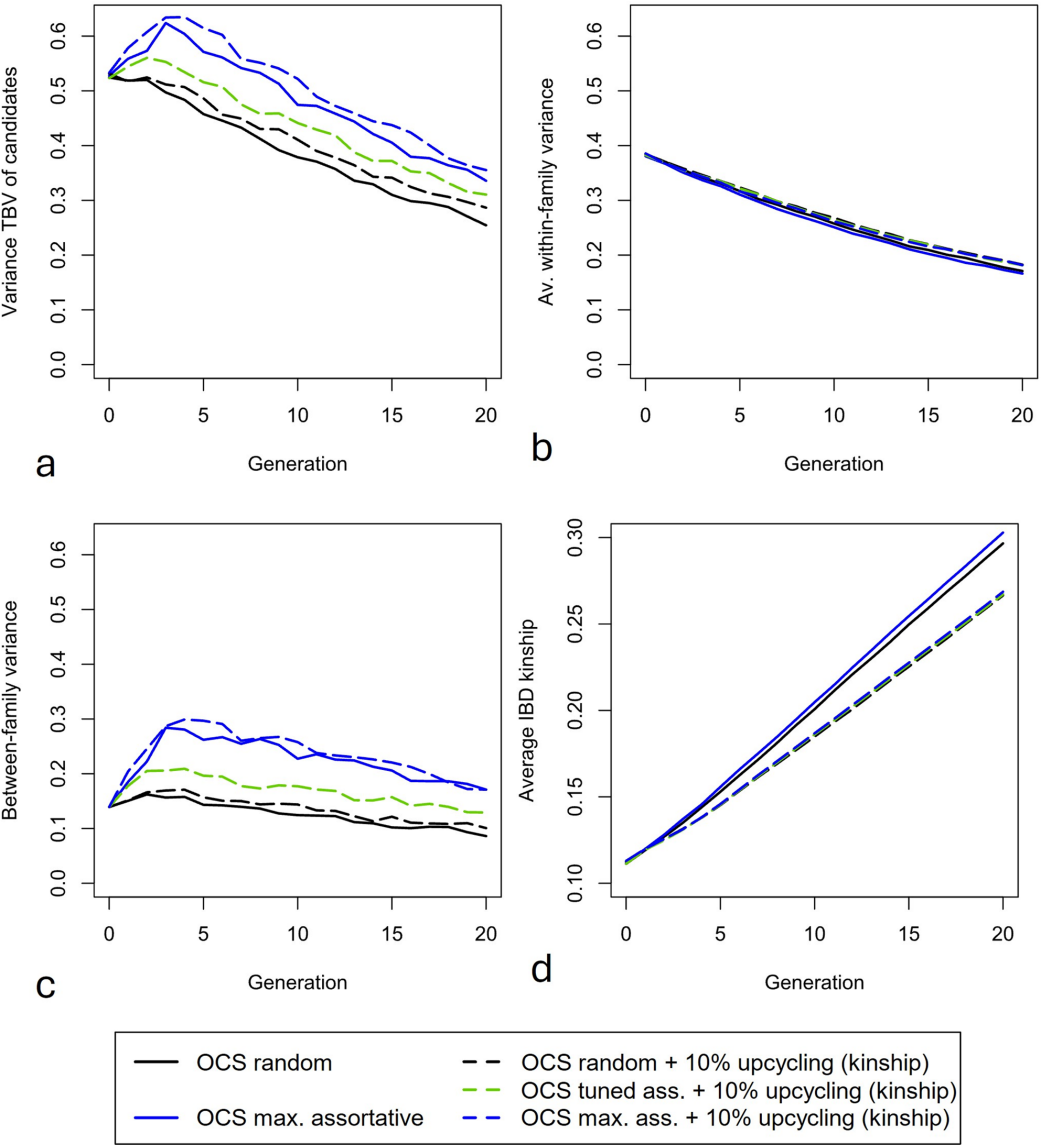
Decomposition of variance of breeding values of selection candidates into between-family variance and within-family variance, and average kinship. Variance of breeding values of elite selection candidates (**a**) as well as the average within-family variance (**b**), the parent-average variance (**c**), and average identity-by-descent kinship (**d**)

As in simulation 1, assortative mating resulted in much higher genetic variance among the elite selection candidates (Fig. 5). The genetic variance in a population is the sum of the parent-average breeding value variance, or the between-family variance, and the average within-family variance (Eq. (1)). Figure 5a shows that differences in total genetic variance arise due to differences in the parent average variance and not due to differences in within-family variances.

The increase in genetic variance by a mating strategy did not influence the development of the kinship level (Fig. 5). Scenarios with diversity introduction showed much lower inbreeding rates than scenarios without diversity introduction. Inbreeding rates were comparable across all mating strategies (~ 0.78%) when diversity introduction was applied. Generally, comparable to simulation 1, the mating strategy did not affect the inbreeding rate much, as it was controlled by OCS. As in [2], breeding programs with diversity introduction were more efficient in turning the loss of diversity into genetic gain. Random mating and maximum assortative mating achieved a genetic gain of 0.82 and 0.84 genetic standard deviations per percentage point increase of kinship level. Breeding programs investing 10% of resources in diversity introduction on average converted 1% point increase in kinship into genetic gains of 0.95, 0.93, and 0.92 units genetic standard deviation.

**Fig. 6:**
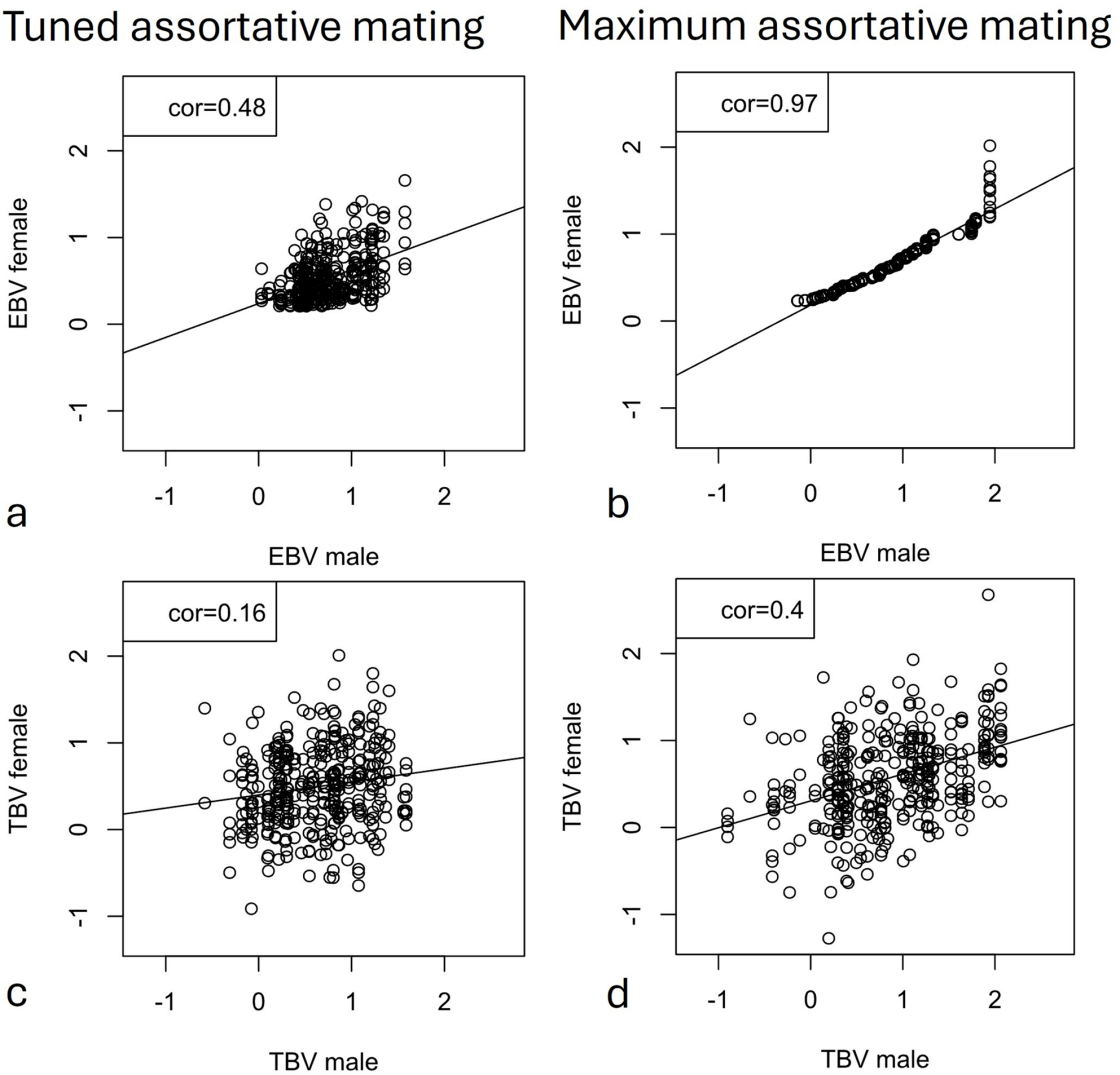
Relationship between estimated and true breeding values of males and their allocated mates under tuned and maximum possible assortative mating. Relationship between estimated breeding values (EBV) of males and those of the females they are mated to (**a** and** b**) and the resulting relationship between their underlying true breeding values (TBV) (**c** and** d**). Shown are values of a random replicate in the last generation of the breeding program for a scheme using tuned assortative mating and a scheme aiming to realize the highest degree of assortative mating. Breeding values are centered to the mean breeding value of elite selection candidates. Pearson correlations are indicated in the plots. The solid black line is the regression line of the linear regression of the breeding value of females on the breeding values of males

To achieve a certain correlation of TBVs between males and their mates, the corresponding correlation of their EBVs must be even higher (Fig. 6, compare a with b and c with d in Additional File 1 Fig. S1). For example, to achieve TBV correlations of 0.15 and 0.37 for the tuned and maximum strategy in generation 20, matings had to be allocated such that the correlation of EBVs was 0.47 and 0.96 (Fig. 6). Also note that the range of EBVs was more reduced due to selection than the range of TBVs. The correlation of EBVs of males and their mates was higher in the generation in which they were selected than one generation later, i.e., after the next genomic evaluation, which included phenotypes of their offspring (compare a with c in Additional File 1 Fig. S1). For example, when mates were allocated in generation 19, the required correlation between EBVs of sires and dams was 0.47 and 0.96. After phenotypes of their offspring were added to the evaluation in generation 20, the correlation of these past matings dropped to 0.33 and 0.71 for the tuned and maximum strategy, respectively. As expected, the correlation of TBVs did not decrease because adding new information to the prediction model does not affect TBVs. Interestingly, even under random mating, we detected a low but consistently positive correlation (about 0.04) between EBVs of males and their mates after their offspring information was included in the training set. Upon additional tests, this appeared to be specific to situations where each female was mated to the same male multiple times, or when each pregnancy resulted in multiple full sib offspring, as in pigs. More description on this is provided in the Additional File 1 Text S1.

On average, the observed correlations matched the required correlations between TBVs predicted with Eq. (7) well, with no systematic over- or underprediction. For example, the average required correlation between TBV of sires and dams in generation 10 was 0.164, while the average observed, i.e., achieved, correlation was 0.159. There was, however, some deviation around this average (Additional File 1 Fig. S2).

**Fig. 7:**
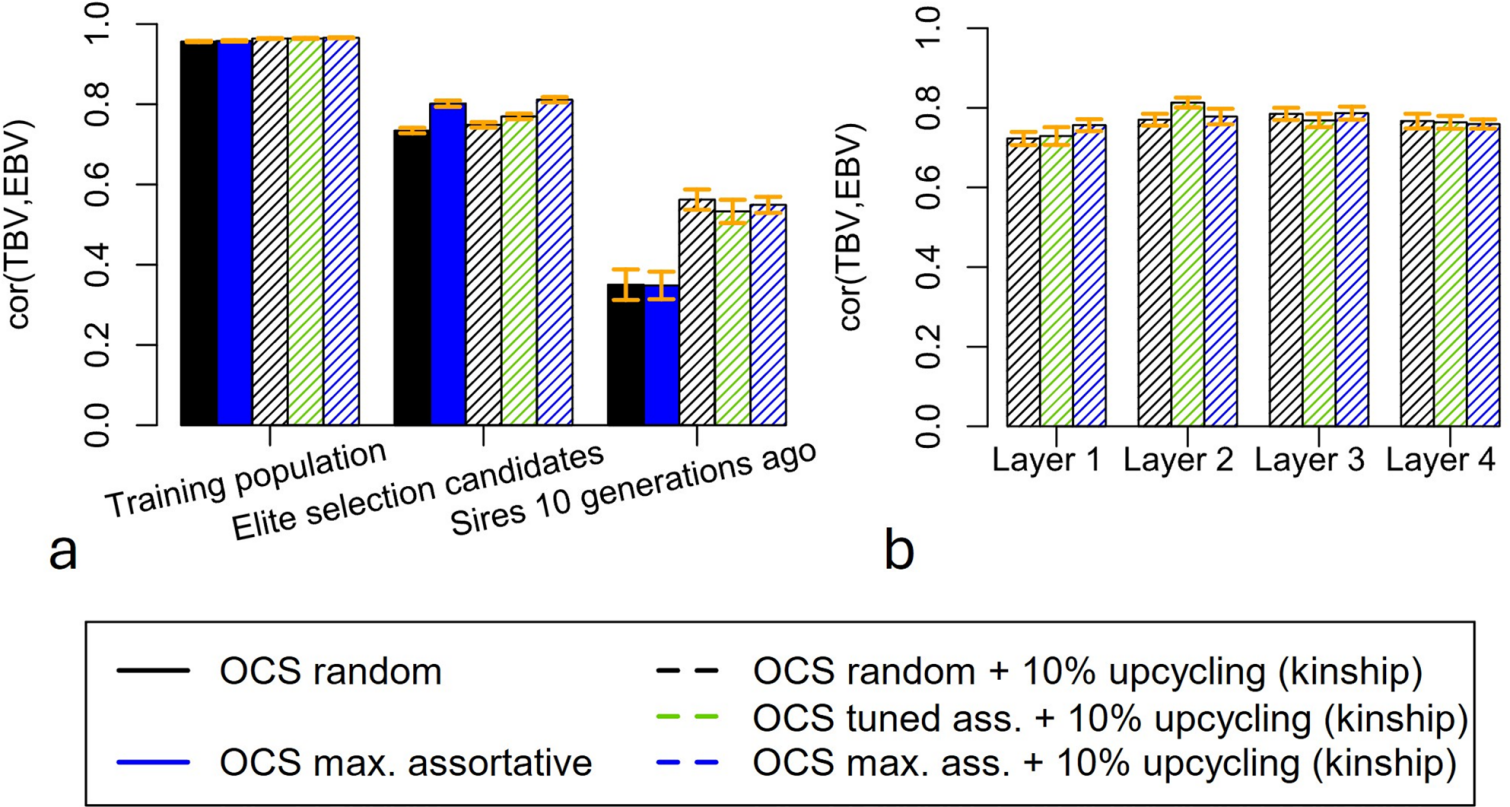
Accuracies of estimated breeding values for different validation groups. The training population, elite selection candidates and sires 10 generations ago (**a**) and the four layers in the upcycling component (**b**). Shown are accuracies in generation 10 of the breeding program. The training population encompasses genotypic and phenotypic information of animals of the current generation and the last 5 generations. Sires 10 generations ago are males that produced elite offspring 10 generations ago. Orange error bars indicate ± the standard error of the mean accuracy of the 20 simulated replicates

As expected, genomic prediction accuracy declined over generations (trend not shown) due to decreasing genetic variance (Fig. 5), resulting in decreasing heritabilities, but the ranking of mating strategies remained unchanged. The smaller elite population size due to diversity introduction, resulting in a smaller proportion of elite animals in the training population, did not result in lower genomic prediction accuracies for elite animals (Fig. 7a). Breeding programs with diversity introduction showed much higher accuracies for EBVs of active sires 10 generations ago, i.e., the oldest sires that were considered as potential diversity donors, than breeding programs without diversity introduction (accuracy approximately 0.57 and 0.35) (Fig. 7). These animals are not included in the genomic evaluation, and also do not have close descendants in the evaluation, unless they were previously selected as diversity donors when they were 9 or fewer generations removed from the elite. Thus, the greater prediction accuracies for the oldest sires with diversity introduction seems to stem from better connectedness of the training population to older generations.

Genomic prediction accuracy in layer 1 of the diversity-introduction component was 0.70, slightly lower than in the elite population (0.75), but increased to approximately 0.77 in the following layers (b in Fig. 7). Thus, own phenotypes and connection to the elite population seemed to outweigh the lower connectedness their donor ancestors have to the training population.

**Fig. 8:**
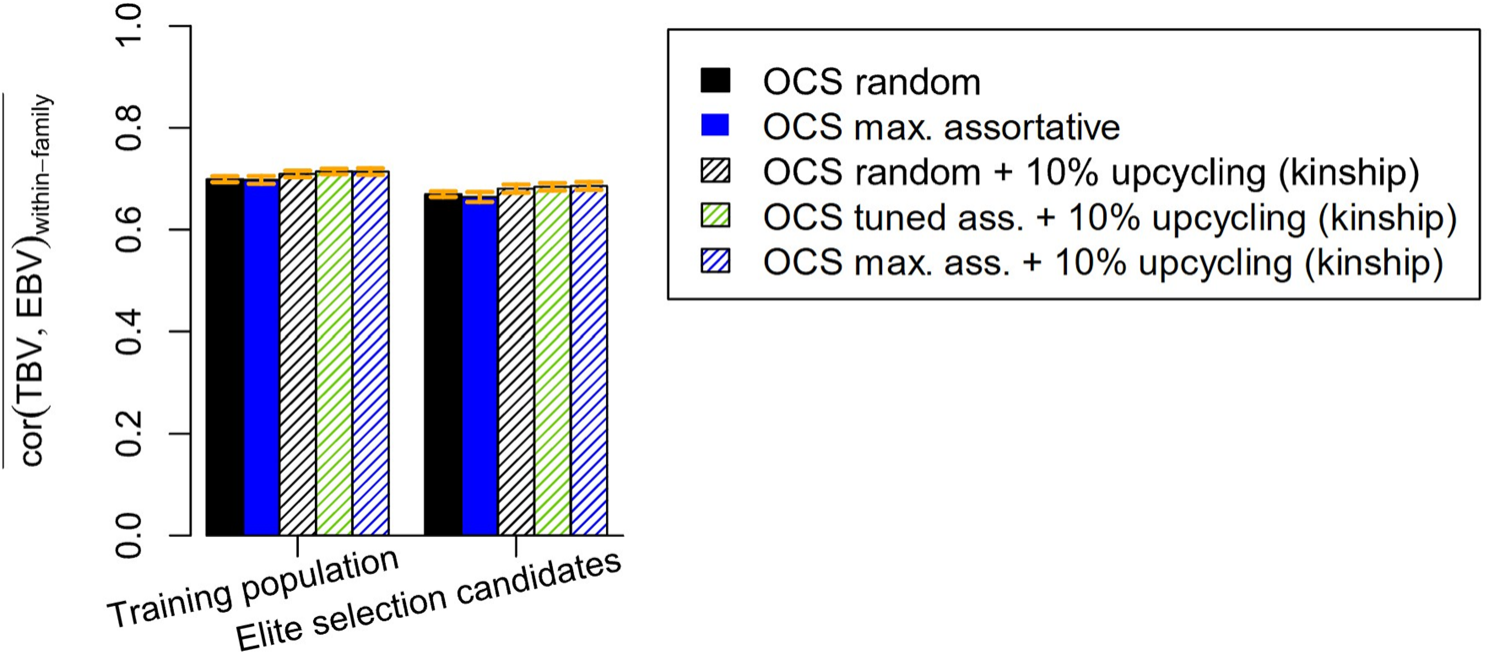
Accuracies of predicted Mendelian sampling terms. Accuracies of Mendelian sampling terms in the training population and in the elite measured as the average within-family correlation between EBVs and TBVs. Shown are accuracies in generation 10 of the breeding program. Orange error bars indicate ± the standard error of the mean accuracy of the 20 simulated replicates

The accuracies of EBVs of elite selection candidates were affected by the mating strategy. For instance, the accuracy was 0.74 for random mating, 0.77 for tuned assortative mating, and 0.81 for maximum assortative mating in generation 10 (Fig. 7). The reason for the increase in accuracy is that assortative mating increased the variance of parent average breeding values (Fig. 5c), which inform the EBVs of offspring to some degree. This is supported by the accuracy within full sib families (Fig. 8) which was very similar across mating strategies. In elite families, it was 0.68 and 0.67 in schemes with and without diversity introduction, respectively. In families in the training population, it was about 0.03 points higher. Hence, the higher accuracy of EBVs on a population level must come from higher accuracy of parent average EBVs.

**Table 1:** Accuracies of EBVs within the group of selected sires and dams in generation 10 (averaged over 20 replicates) and the maximum correlation between TBV of sires and their mates

	Cor(TBV_sire_, EBV_sire_)	Cor(TBV_dam_, EBV_dam_)	Max. pos. cor(TBV_sire_, TBV_dam_)
OCS rand.	0.58	0.48	0.28
OCS max. assortative	0.73	0.60	0.44 (achieved: 0.42)
OCS rand. + 10% upcycling	0.61	0.50	0.31
OCS tuned ass. + 10% upcycling	0.67	0.55	0.36
OCS max. ass. + 10% upcycling	0.74	0.60	0.45 (achieved: 0.44)

Accuracies are adjusted for varying genetic contributions of males to the next generation so that they reflect the correlations needed in the denominator of Eq. (7). The highest achievable correlation between TBVs of sires and dams when mating animals assortatively on EBVs is indicated. It is calculated as cor(EBV_sire_, EBV_dam_)*cor(TBV_sire_, EBV_sire_)*cor(TBV_dam_, EBV_dam_) and assuming cor(EBV_sire_, EBV_dam_) = 1

The accuracy of EBVs calculated for the group of selected sires and dams was lower than the accuracy calculated over all elite animals (compare Table 1; Fig. 7) due to reduction of genetic variance caused by selection. Note that the latter accuracy drives response to selection [3, 21], whereas the former is needed to calculate the required correlation between EBVs for assortative mating (Eq. (7)). Accuracies within the group of selected animals were also affected by the mating strategy. The highest achievable correlation between TBVs of males and their mates was intermediate at 0.45. This shows that the success of an assortative mating strategy is strongly dependent on genomic prediction accuracies. Accuracies within the group of selected animals are only needed for the tuned assortative mating strategy. In this study, they were predicted based on the estimated prediction error variance of the EBVs. The average predicted accuracy was 0.66 within the group of selected sires and 0.56 within the group of selected dams and were on average unbiased estimated of the true accuracies. However, observed accuracies of individual replicates deviated from the prediction, especially when the predicted accuracy was low (below ~ 0.5) (visualized in Additional File 1 Fig. S3). The predicted accuracy may partly be improved by considering covariances between prediction errors in the calculation of the group-genetic variance, which we ignored in this study.

**Fig. 9:**
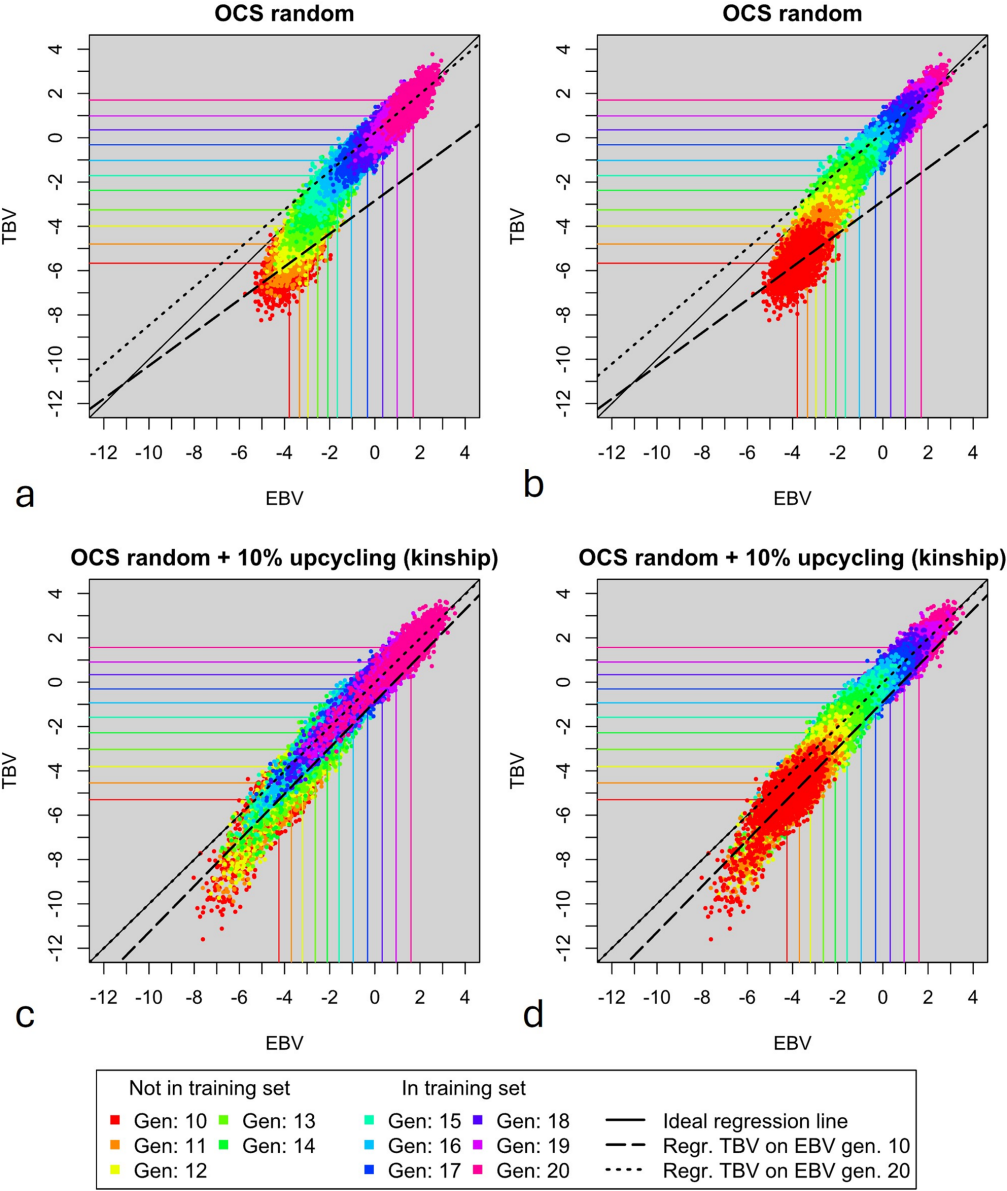
Distribution of estimated and true breeding values of animals of the past 10 generations in breeding schemes with and without diversity introduction. Distribution of TBVs and EBVs for OCS with random mating without diversity introduction (**a**,** b**) and with diversity introduction (**c**,** d**). The plotted values are taken from the last generation of the random replicates. Plots **b** and **d** are identical to plots **a** and **c** with the difference that animals of older generations were drawn last to convey better intuition of the distribution on older generations. Breeding values of generations 10 to 14 are predicted indirectly based on estimated SNP effects estimated based on data of generations 15 to 20. TBVs and EBVs were centered by the average TBV and EBV of generations 15 to 20. Colored lines indicate the average TBV and EBV for the respective generation

Figure 9 shows that the main effect the upcycling component had was increasing the range of TBV within a generation, with animals in early layers on the low end and elite animals on the high end. In addition, genomic predictions of older generations were less biased in upcycling schemes. In comparison to the upcycling component, the mating strategy had a much smaller effect and distributions of them are thus not included in the figure.

**Table 2:** Level bias for the training set, elite animals, active sires 10 generations ago, and animals born from Donor x Elite matings (Layer 1)

	Training set	Elite	Sires 10 generations ago	Layer 1	Estimated genetic standard deviation
OCS random	−0.004 (0.051)	−0.004 (0.015)	−2.001 (0.062)	/	0.683 (0.014)
OCS max. assortative	−0.004 (0.063)	−0.008 (0.014)	−2.941 (0.063)	/	0.686 (0.015)
OCS random + 10% upcycling (kinship)	−0.005 (0.047)	−0.020 (0.014)	−0.785 (0.040)	−0.315 (0.039)	0.708 (0.012)
OCS tuned ass. + 10% upcycling (kinship)	−0.005 (0.050)	−0.025 (0.015)	−0.781 (0.046)	-0.347 (0.038)	0.708 (0.014)
OCS max. ass. + 10% upcycling (kinship)	−0.004 (0.045)	−0.022 (0.014)	−0.734 (0.039)	-0.297 (0.046)	0.714 (0.015)

Level bias is the absolute difference between true and estimated breeding values expressed in units of estimated genetic standard deviations ($\:{\widehat{\upsigma\:}}_{A}$). A negative difference means EVBs overestimate TBVs on average. If there is no bias, the difference is 0.

Shown are averages in generation 10 of the breeding program. Values in parentheses are standard errors.

Breeding programs without diversity introduction showed lower accuracies for genomic predictions of older generations (Fig. 7) and overestimated (negative level bias) the TBV of past generations that were not included in the breeding value evaluation (Fig. 9; Table 2). Breeding programs with and without diversity introduction showed inflation of the variance of EBVs for old sires that were not included in the evaluation, i.e., the slope of the regression of TBVs on EBVs was less than 1. This dispersion bias was less pronounced in diversity introduction schemes (Table 3). Breeding values of animals that were born into layer 1 as the result of Donor x Elite matings showed more level bias and dispersion bias than elite animals. The lower biases of old sires for breeding programs with diversity introduction may be explained by better genetic connectedness to the training population. The mating strategy did not appear to cause more or less bias compared to random mating, except for the group of old sires (Tables 2 and 3).

**Table 3:** Dispersion bias for the training set, elite animals, active sires 10 generations ago, and animals born from Donor x Elite matings (Layer 1)

	Training set	Elite	Sires 10 generations ago	Layer 1
OCS random	0.99 (0.002)	0.90 (0.008)	0.57 (0.044)	/
OCS max. assortative	0.99 (0.003)	0.93 (0.010)	0.73 (0.038)	/
OCS random + 10% upcycling (kinship)	1.01 (0.003)	0.90 (0.009)	0.63 (0.026)	0.85 (0.033)
OCS tuned ass. + 10% upcycling (kinship)	1.01 (0.004)	0.90 (0.009)	0.68 (0.023)	0.80 (0.034)
OCS max. ass. + 10% upcycling (kinship)	1.01 (0.004)	0.91 (0.009)	0.71 (0.028)	0.84 (0.035)

Dispersion bias is the slope of the regression of TBVs on EBVs. A value lower than 1 means that the variance of EBVs is relatively inflated, i.e., a change of 1 unit on the EBV scale results in less than 1 unit change on the TBV scale. Shown are averages in generation 10 of the breeding program. Standard errors are given in parentheses.

## Discussion

The aims of this study were to examine effects of assortative mating on genetic gain, genetic merit of AI sires, genetic diversity, and prediction accuracy achieved with genomic selection and test its benefits in a multi-layer pig breeding program with diversity introduction, using stochastic simulation. Without any form of inbreeding control, assortative mating yielded commercial AI boars that were consistently better than with random or disassortative mating. Assortative mating resulted in a population that was at most 0.6 and 0.8 genetic standard deviations ahead of a population with, respectively, random and disassortative mating. This slight increase in genetic gain was at the expense of a very high rate of inbreeding of 2.2% per generation and associated higher loss of genetic variance per generation, which is in line with previous findings [8–11]. The elevated inbreeding rate caused a faster loss of genetic variance in the long-term, which eventually resulted in a decrease in the genetic merit shown by assortative mating populations compared to random mating populations (decreasing gap of level after the maximum difference of 0.6 gSD was reached, Fig. 2). As described earlier, the elevated inbreeding rate with truncation selection in populations created with assortative mating is due to the larger variance of contributions of families to the next generation [3, p. 67–69], which is due to the larger variance of family means resulting from assortative mating (Figs. 1 and 5). Disassortative and random mating had approximately the same rate of inbreeding and loss of genetic variance. With OCS with a target rate of inbreeding of 1% per generation, the inbreeding rates achieved with different mating strategies were comparable. Improved commercial gain achieved when assortative mating was coupled with OCS agrees with results of Rosvall and Mullin [14] for forest tree breeding. In our set-up with diversity introduction, assortative mating was not able to fully compensate for the slower genetic gain over multiple generations because part of the elite population resources were dedicated to upcycling.

The simulated truncation selection scenario did not employ any strategy for inbreeding control. Practical breeding programs typically employ some sort of inbreeding control and might consider more criteria than just the total merit selection index or GEBV. Similarly, OCS represents a case with optimal inbreeding control that might also not be perfectly attainable in practice. We compared truncation selection (no inbreeding control) against OCS for a maximum contrast. With this in mind, the findings of our study show that inbreeding control becomes more important when using assortative mating.

Genetic variance is increased by assortative mating under OCS selection because assortative mating increases the between-family variance but not the within-family variance (Fig. 5). Under the infinitesimal model, the latter is only affected by the inbreeding level of the parents ($\:{\upsigma\:}_{within}^{2}=0.25\left(1-{F}_{sire}\right){\upsigma\:}_{base}^{2}+0.25\left(1-{F}_{dam}\right)0.25{\upsigma\:}_{base}^{2}$) and the inbreeding rate is controlled by OCS. Thus, assortative mating helped to improve the competitiveness of breeding schemes with diversity introduction in the short-term (up to 5 generations) (Fig. 4). Yet, in the long-term, the genetic level of breeding programs with diversity introduction, even the commercial genetic level, was lower than that of breeding programs not investing in diversity introduction, due to the smaller size of the elite population.

Generally, the amount by which genetic variance can be increased by assortative mating depends on the accuracy of EBVs (Eq. (5)) and the genetic variance among the selected animals (Eq. (7)), which is influenced by the proportion selected [7, p. 569–572]. The benefit of assortative over random mating regarding the increase of genetic variance is greatest with lower selection intensity. Although genomic prediction generally achieves higher prediction accuracies than predictions based on pedigree or phenotypes alone, we show that the maximum achievable correlation between TBVs of assortatively mated sires and dams is at most intermediate, even with genomic prediction (Table 1). This is because the accuracy of EBV, i.e., the correlation between TBV and EBV, is reduced within the group of selected males and females due to selection-induced reduction of variance of EBVs.

Contrary to our expectations, the genomic prediction model did not appear to estimate SNP effects less accurately under assortative mating or when reducing the elite population size to accommodate diversity introduction (Fig. 8). The expected reduction in accuracies, was, however, the reason for us to design the tuned assortative mating strategy to reduce potential disadvantages as much as possible. Our findings show that there is little harm in using assortative mating in genomic selection programs, as long as genetic diversity can be controlled reliably, e.g., with OCS and appropriate diversity metrics. Thus, if the inbreeding rate is under control, breeding program managers are advised to use the maximum assortative mating strategy instead of our tuned strategy.

It is good to note that accuracies of selection in the population improvement component may be reduced if, contrary to our design, only offspring of top animals or AI animals are phenotyped and used to train the prediction model. Such a phenotyping scheme would reduce the connection between selection candidates and the animals with phenotypes, e.g. animals on commercial farms, and consequently reduce the accuracy for selection candidates, as shown in Figure 5 of Wientjes et al. [22]. Since AI animals are selected from fewer families under assortative mating schemes compared to random mating ones (Fig. 1), the phenotypes would be informative for selection candidates of these families. But these phenotypes may be less informative for all other families that did not produce offspring in commercial environments and are, thus, expected to be less related to the training population [23–25]. In addition, we did not find reduced accuracies when part of the genomic prediction training set has diversity-donor-ancestry, in line with findings of Allier et al. [26], i.e., mixing donor and elite does not come at the expense of elite accuracies. This may be because we used donors from the same breeding line. If donors are from other lines or breeds are used, the accuracy might be impacted. Note that our implementation of the upcycling scheme does not need EBVs for animals in early layers since they are solely selected based on having a low average kinship to the elite population. Thus, if a system is in place that keeps the introduced diversity in the population, optimizing the training set design will be less important as the GEBV is not the sole criterion for selection. The optimal design of the genomic prediction training population when diversity is to be introduced into the elite population requires further study.

For simplicity, we treated selection and mating as independent subsequent steps in this study. Potentially more benefits may be realized by solving the mating and selection problems simultaneously. An idea how to go about this is described in more detail in Additional File 1 Text S2.

The intention for the use of an assortative mating strategy in a breeding program was to overcome the guaranteed short-term losses in population genetic gain associated with the introduction of diversity. The hypothesis was that diversity flowing in through the upcycling layers would give the elite population a competitive edge, which would improve long-term genetic gain. However, this was not observed in our simulation because OCS, as a strategy to control inbreeding, results in minimal loss of favorable alleles, so introduced diversity has less to ‘repair’. Thus, anything that deviates from optimal inbreeding control can be assumed inferior with regard to management of favorable alleles. Thus, the upcycling strategy and assortative mating may be beneficial if the population is not selected with OCS, experienced a bottleneck in the past, or if the breeding goal changed between the time that the diversity donors were alive and the current generation.

## Conclusion

Assortative mating is a promising method to increase between-family variance and thereby increasing commercial genetic gain if the inbreeding rate can be controlled e.g. with OCS. Under truncation selection without inbreeding control, assortative mating increases the rate of inbreeding, which leads to faster depletion of genetic diversity and thus limits long-term genetic gain. Assortative mating could increase the genetic merit of AI sires of breeding programs if the nucleus breeding population size is reduced due to using resources for diversity introduction, but this benefit was not large enough to outperform a competitor that did not have reduced nucleus size. We developed a tuned assortative mating strategy to mitigate negative effects we expected from assortative mating, e.g. a decrease in the accuracy of genomic predictions. However, genomic prediction accuracy measured as within-family accuracy was not impacted by the mating strategy and, thus, the tuned strategy was not necessary. The developed equations, however, show that the impact of assortative mating on genetic variance in the next generation decreases when the accuracy of EBV and the genetic variance among selected parents decreases, i.e., when the selection intensity increases.

## Supplementary Information

Below is the link to the electronic supplementary material.

Additional file 1: Supplementary Fig. S1, S2 and S3. The file also contains detailed description of positive correlation between EBVs of males and females they were mated to under random mating, and explanation of this phenomenon with larger full sib groups (Text S1). The file also contains a detailed description of an idea to combine the mate allocation and selection problem (Text S2).

Additional file 2: Simple simulation with OCS, selection, variance component estimation and tuned assortative mating. This script shows the steps we took to obtain the required correlation between EBVs of males and females to achieve a certain genetic variance in the next generation. It ends with checking if indeed the achieved correlation of TBVs matches the required one. Needs the population in vcf format provided in Additional file 5 and the SNP information provided in Additional file 6 as input. Also, functions defined in Additional file 3 are required.

Additional file 3: This R script contains function definitions needed by Additional file 2.

Additional file 4: This R script contains code showing how we simulated the population history. The last generation generated in this script is used as input for Additional file 2. An example of such an output is provided in Additional file 5.

Additional file 5: This vcf file is the output of the population history simulation from Additional file 4. It is needed for Additional file 2.

Additional file 6: This text file is needed to initiate the population obtained from population history simulation with Additional file 4. It is generated from Additional file 4. It stores information for all SNPs with regard to whether they are used for the genotyping array, or carry QTL effects. This file is needed for simulation in Additional file 2.

Additional file 7: This R script shows the steps taken in the mate allocation algorithm.

## Data Availability

The datasets generated and/or analyzed during the current study are not publicly available but are available from the corresponding authors on reasonable request. R scripts to test the presented methods are provided in additional files.

## Appendix

**Detailed derivation of required correlation between breeding values of sires and dams**.

In this section, we will explain how to work out the genetic variance needed so that the animals selected as AI boars have the same genetic level as a competitor breeding program, given that the selection intensity should not be changed. This is needed to derive the correlation between EBVs of sires and the dams they should be mated to. This correlation is needed for our “tuned assortative mating strategy”.

We explain how to derive the variance components needed for Eq. (1) in the following. It will be explained for the sires only but the calculation for the dams is identical. To derive the variance of TBVs of selected males, the variance of the prediction errors $\mathrm{(var(TBV-EBV)}$; $\overline{{{\mathrm{PEV}}}} _{{\mathrm{sires}}}$) needs to be considered in Eq. (8).

8 $$ \upsigma _{{{\mathrm{TBV}}\:{\mathrm{sires}}}}^{2} = \upsigma _{{{\mathrm{EBV}}\:{\mathrm{sires}}}}^{2} + \overline{{{\mathrm{PEV}}}} _{{{\mathrm{sires}}}} . $$

Variation of genetic contributions to the next generation among males also needs to be accounted for in the variance prediction. This can be done with Eq. (9) for the term $\upsigma _{{{\mathrm{EBV}}\:{\mathrm{sires}}}}^{2}$ and Eq. (10) for the term $\overline{{{\mathrm{PEV}}}} _{{\mathrm{sires}}}$. The average EBV weighted by the contributions required by Eq. (9) can be obtained with Eq. (11). Equation (10) requires the genetic variance that was estimated in the (hypothetical) unselected, non-inbred and randomly mating base generation ($\upsigma _{{{\mathrm{A}}_{{{\mathrm{base}}}} }}^{2}$), as well as the reliability for every individual, which is estimated (${\widehat{\mathrm{r}}}_{{\mathrm{E}\mathrm{B}\mathrm{V},\:\mathrm{T}\mathrm{B}\mathrm{V}}_{\mathrm{s}\mathrm{i}\mathrm{r}\mathrm{e}\:\mathrm{i}}}^{2}$). In this study, $\upsigma _{{{\mathrm{A}}_{{{\mathrm{base}}}} }}^{2}$ was estimated with the R package rrBLUP [19] using 3000 random elite animals sampled from the current and the last three generations. $\upsigma _{{A_{{base}} }}^{2}$ and heritability were estimated every third generation using a genomic relationship matrix as in Niehoff et al. [2]. In this study, the reliabilities of individuals (${\widehat{\mathrm{r}}}_{{\mathrm{E}\mathrm{B}\mathrm{V},\:\mathrm{T}\mathrm{B}\mathrm{V}}_{\mathrm{s}\mathrm{i}\mathrm{r}\mathrm{e}\:\mathrm{i}}}^{2}$) were estimated using the method presented in VanRaden [28] and implemented in MoBPS. The model-estimated individual reliability is the square of the accuracy in the base population which allows calculating the prediction error variance as ${\mathrm{P}\mathrm{E}\mathrm{V}}_{\mathrm{i}}=\left(1-{\widehat{\mathrm{r}}}_{{\mathrm{E}\mathrm{B}\mathrm{V},\:\mathrm{T}\mathrm{B}\mathrm{V}}_{\mathrm{s}\mathrm{i}\mathrm{r}\mathrm{e}\:\mathrm{i}}}^{2}\right)\text{}{{\upsigma\:}}_{{\mathrm{A}}_{\mathrm{b}\mathrm{a}\mathrm{s}\mathrm{e}}}^{2}$ [29]. That reliability measure is not the same as the squared correlation of EBV and TBV due to the selection-induced reduction of genetic variance [21, 29].

9 $$\upsigma _{{{\mathrm{EBV}}\:{\mathrm{sires}}}}^{2} = \sum\nolimits_{{{\mathrm{i}} = 1}}^{{{\mathrm{n}}\:{\mathrm{sires}}}} {{\mathrm{contribution}}_{{{\mathrm{sire}}\:{\mathrm{i}}}} {\text{}}\left( {{\mathrm{EBV}}_{{{\mathrm{sire}}\:{\mathrm{i}}}} - \overline{{{\mathrm{EBV}}}} _{{{\mathrm{sire}}}} } \right)^{2} } $$

10 $$\overline{{{\mathrm{PEV}}}} _{{{\mathrm{sires}}}} = \sum\nolimits_{{{\mathrm{i}} = 1}}^{{{\mathrm{n}}\:{\mathrm{sires}}}} {\left( {1 - \widehat{{\mathrm{r}}}_{{{\mathrm{EBV}},\:{\mathrm{TBV}}_{{{\mathrm{sire}}{\kern 1pt} {\mathrm{i}}}} }}^{2} } \right)} {\text{}}\upsigma _{{{\mathrm{A}}_{{{\mathrm{base}}}} }}^{2} {\text{}}\frac{{{\mathrm{contribution}}_{{{\mathrm{sire}}\:{\mathrm{i}}}} }}{{\sum\nolimits_{{{\mathrm{i}} = 1}}^{{{\mathrm{n}}\:{\mathrm{sires}}}} {{\mathrm{contribution}}_{{{\mathrm{sire}}\:{\mathrm{i}}}} } }} $$

11 $$\overline{{{\mathrm{EBV}}}} _{{{\mathrm{sires}}}} = \sum\nolimits_{{{\mathrm{i}} = 1}}^{{{\mathrm{n}}\:{\mathrm{sires}}}} {\frac{{{\mathrm{contribution}}_{{{\mathrm{sire}}\:{\mathrm{i}}}} }}{{\sum {\:_{{{\mathrm{i}} = 1}}^{{{\mathrm{n}}\:{\mathrm{sires}}}} } {\mathrm{contribution}}_{{{\mathrm{sire}}\:{\mathrm{i}}}} }}{\mathrm{EBV}}_{{{\mathrm{sire}}\:{\mathrm{i}}}} } $$

The variance of Mendelian sampling terms passed on to offspring by an individual can be predicted based on the inbreeding level of the animal as $\upsigma _{{{\mathrm{gamMS}}}}^{2} = \left( {1 - {\mathrm{F}}_{{{\mathrm{ind}}}} } \right){\text{}}\upsigma _{{{\mathrm{A}}_{{{\mathrm{base}}}} }}^{2} {\text{}}0.25$ [30] under the assumptions of the infinitesimal model. Since the base generation shows some level of inbreeding, we use the difference between the homozygosity of an animal and the average inbreeding level in the base generation. The average inbreeding level in the base generation (${F}_{base}$) was obtained as the average kinship level of the 3000 animals used for variance component estimation. The weighted average gametic MSV of males was calculated with Eq. (12).

12 $$\begin{aligned} \overline{\upsigma } _{{{\mathrm{gametic}}\:{\mathrm{MS}}\:{\mathrm{sires}}}}^{2} = & \sum\nolimits_{{{\mathrm{i}} = 1}}^{{{\mathrm{n}}\:{\mathrm{sires}}}} {\left( {1 - \left( {{\mathrm{F}}_{{{\mathrm{sire}}\:{\mathrm{i}}}} - {\mathrm{F}}_{{{\mathrm{base}}}} } \right)} \right)} \\ & {\text{}}\frac{{\upsigma _{{{\mathrm{A}}_{{{\mathrm{base}}}} }}^{2} }}{4}{\text{}}\frac{{{\mathrm{contribution}}_{{{\mathrm{sire}}\:{\mathrm{i}}}} }}{{\sum\nolimits_{{{\mathrm{i}} = 1}}^{{{\mathrm{n}}\:{\mathrm{sires}}}} {{\mathrm{contribution}}_{{{\mathrm{sire}}\:{\mathrm{i}}}} } }} \\ \end{aligned} $$

We assume that the competitor breeding program is mating the selected animals at random, such that ${C}\mathrm{O}\mathrm{V}\left({\mathrm{T}\mathrm{B}\mathrm{V}}_{{\mathrm{s}\mathrm{i}\mathrm{r}\mathrm{e}\mathrm{s}}_{\mathrm{t}}},{\mathrm{T}\mathrm{B}\mathrm{V}}_{{\mathrm{d}\mathrm{a}\mathrm{m}\mathrm{s}}_{\mathrm{t}}}\right)=0$, and we here assume that the breeding program of the competitor is equivalent to the un-layered version of the own breeding program. Under these assumptions, we can predict the genetic level of AI boars of the competitor in the next generation with Eq. (13).

13 $$\begin{aligned} \overline{{{\mathrm{TBV}}}} _{{{\mathrm{AI}}\:{\mathrm{competitor}}}} = & {\mathrm{PA}}_{{{\mathrm{EBV}}\:{\mathrm{competitor}}}} + {\mathrm{i}}_{{{\mathrm{competitor}}}} \\ & {\mathrm{r}}_{{{\mathrm{TBV}},{\mathrm{EBV}}_{{{\mathrm{cand}}}} }} {\text{}}\upsigma _{{{\mathrm{TBV}}_{{{\mathrm{competitor}}}} }} \\ \end{aligned} $$

The standard deviation of TBVs in the next generation of the competitor breeding program (${{\upsigma}}_{{\mathrm{T}\mathrm{B}\mathrm{V}}_{\mathrm{c}\mathrm{o}\mathrm{m}\mathrm{p}\mathrm{e}\mathrm{t}\mathrm{i}\mathrm{t}\mathrm{o}\mathrm{r}}})$ can be calculated by summing the variance of TBVs, which itself is the sum of the variance of EBVs and the average prediction error variance, and the gametic Mendelian Sampling variances of sires and dams (Eq. (1)), and taking the square root of the result. In Eq. (13), $\overline{{{\mathrm{TBV}}}} _{{\text{AI competitor}}}$ is the average TBV of AI boars, ${\mathrm{P}\mathrm{A}}_{\mathrm{E}\mathrm{B}\mathrm{V}\:\mathrm{c}\mathrm{o}\mathrm{m}\mathrm{p}\mathrm{e}\mathrm{t}\mathrm{i}\mathrm{t}\mathrm{o}\mathrm{r}}$ is the parent average EBV of selected animals, which is identical to their average TBV since the regression of TBVs on EBVs in 1, ${\mathrm{i}}_{\mathrm{c}\mathrm{o}\mathrm{m}\mathrm{p}\mathrm{e}\mathrm{t}\mathrm{i}\mathrm{t}\mathrm{o}\mathrm{r}}$ is the selection intensity used by the competitor, ${\mathrm{r}}_{{\mathrm{T}\mathrm{B}\mathrm{V},\mathrm{E}\mathrm{B}\mathrm{V}}_{\mathrm{c}\mathrm{a}\mathrm{n}\mathrm{d}}}$is the genomic prediction accuracy, i.e., the Pearson correlation between TBVs and EBVs of candidates, and ${{ \upsigma }}_{{\mathrm{T}\mathrm{B}\mathrm{V}}_{\mathrm{c}\mathrm{o}\mathrm{m}\mathrm{p}\mathrm{e}\mathrm{t}\mathrm{i}\mathrm{t}\mathrm{o}\mathrm{r}}}$ is the standard deviation of TBVs in the next generation of the competitor. We assume that the accuracy in the next generation is identical to the accuracy in the current generation. The expectation of this accuracy can be obtained with the prediction error variances predicted based on reliabilities of candidates and the variance of EBVs (Eqs. (14) and (15)). We only considered elite males as candidates for the calculation of prediction error variance and variance of EBVs.

14 $${\mathrm{P}\mathrm{E}\mathrm{V}}_{\mathrm{c}\mathrm{a}\mathrm{n}\mathrm{d}\:\mathrm{i}}=\left(1-{\widehat{\mathrm{r}}}_{{\mathrm{E}\mathrm{B}\mathrm{V},\:\mathrm{T}\mathrm{B}\mathrm{V}}_{\mathrm{c}\mathrm{a}\mathrm{n}\mathrm{d}\:\mathrm{i}}}^{2}\right)\text{}{{\upsigma\:}}_{{\mathrm{A}}_{\mathrm{b}\mathrm{a}\mathrm{s}\mathrm{e}}}^{2}$$

15 $${\mathrm{r}}_{{{\mathrm{TBV}},{\mathrm{EBV}}_{{{\mathrm{cand}}}} }} = \frac{1}{{{\mathrm{n}}\:{\mathrm{candidates}}}}{\text{}}\sum\nolimits_{{{\mathrm{i}} = 1}}^{{{\mathrm{n}}\:{\mathrm{candidates}}}} {\sqrt {1 - \frac{{{\mathrm{PEV}}_{{{\mathrm{cand}}\:{\mathrm{i}}}} }}{{\upsigma _{{{\mathrm{EBV}}_{{{\mathrm{cand}}}} }}^{2} + {\mathrm{PEV}}_{{{\mathrm{cand}}\:{\mathrm{i}}}} }}} } $$

We assume that the competitor selects the same animals as the own breeding program, which means the parent average breeding values are the same (${\mathrm{P}\mathrm{A}}_{\mathrm{E}\mathrm{B}\mathrm{V}\:\mathrm{c}\mathrm{o}\mathrm{m}\mathrm{p}\mathrm{e}\mathrm{t}\mathrm{i}\mathrm{t}\mathrm{o}\mathrm{r}}={\mathrm{P}\mathrm{A}}_{\mathrm{E}\mathrm{B}\mathrm{V}\:\mathrm{o}\mathrm{w}\mathrm{n}}$). We further assume that the selection accuracy (${\mathrm{r}}_{{{\mathrm{TBV}},\:{\mathrm{EBV}}_{{{\mathrm{cand}}}} }}$) of the competitor is the same as the own accuracy.

The selection intensity of the competitor breeding program (${\mathrm{i}}_{\mathrm{c}\mathrm{o}\mathrm{m}\mathrm{p}\mathrm{e}\mathrm{t}\mathrm{i}\mathrm{t}\mathrm{o}\mathrm{r}}$) can be derived from the selection proportion. For example, say the competitor selects 80 out of 1200 male candidates and the own breeding program needs to select 80 out of 1080 candidates, then ${\mathrm{i}}_{\mathrm{c}\mathrm{o}\mathrm{m}\mathrm{p}\mathrm{e}\mathrm{t}\mathrm{i}\mathrm{t}\mathrm{o}\mathrm{r}}=\mathrm{i}\left(\mathrm{p}=\frac{80}{1200}\right)=1.94$ and ${\mathrm{i}}_{\mathrm{o}\mathrm{w}\mathrm{n}}=\mathrm{i}\left(\mathrm{p}=\frac{80}{1080}\right)=1.89$. The selection intensity can be calculated in R as: dnorm(qnorm(1-p))/p. The selection intensity expresses in genetic standard deviations units the difference between the mean of selected animals and the average of the population. Given that the number of AI sires a breeding program needs every generation is the same, i.e., the own breeding program cannot lower the selected proportion to make up for the smaller elite population size, we assume that the selection intensity remains unchanged. Instead, the unit, the standard deviation, can be adjusted. The standard deviation of EBVs that is required in the next generation to match the competitor can be calculated with Eq. (2). Thus, the required variance of TBVs in the next generation for the own breeding program can be calculated (Eq. (3)).

Given that we know the variance of TBV in our own breeding program under random mating (Eq. (16)), we can derive the required covariance between TBVs of males and females that are to be mated (Eq. (4)), and thus also the correlation between them (Eq (5)). With Eq. (7), the required correlation between sire and dam EBVs can be calculated and subsequently be used to construct a mating plan. Note that the accuracies in Eqs. (6) and (7) (${\mathrm{r}}_{{\mathrm{E}\mathrm{B}\mathrm{V},\mathrm{T}\mathrm{B}\mathrm{V}}_{\mathrm{s}\mathrm{i}\mathrm{r}\mathrm{e}\mathrm{s}}},\:{\mathrm{r}}_{{\mathrm{E}\mathrm{B}\mathrm{V},\mathrm{T}\mathrm{B}\mathrm{V}}_{\mathrm{d}\mathrm{a}\mathrm{m}\mathrm{s}}}$) refer to the correlation of TBV and EBV in the group of selected sires and dams and are thus not identical to ${\mathrm{r}}_{{\mathrm{T}\mathrm{B}\mathrm{V},\mathrm{E}\mathrm{B}\mathrm{V}}_{\mathrm{c}\mathrm{a}\mathrm{n}\mathrm{d}}}$. Since selection reduces the genetic variance within the group of selected animals, the accuracy within the group of selected animals is reduced compared to the accuracy among all candidates. An R script showing the implementation of tuned assortative mating following OCS in MoBPS can be found in Supplementary Material 2 Code S1. That R script requires functions that are defined in an R script in Supplementary Material 3 Code S2. Supplementary Material 4 Code S3 simulates population history and writes out a vcf file with animals to use as a start population and SNP information for Supplementary Material 3 Code S4. An exemplary output of population history simulation is provided in Supplementary Materials 6 Code S5 and 7 Code S6 to enable testing right away.

16 $$ \begin{aligned} \upsigma _{{{\mathrm{TBV}}_{{{\mathrm{own~rand~t}} + 1}} }}^{2} = & \frac{{\upsigma _{{{\mathrm{EBV}}_{{{\mathrm{sires}} }} }}^{2} + \overline{{{\mathrm{PEV}}}} _{{{\mathrm{sires}}}} }}{4} \\ & + \frac{{\upsigma _{{{\mathrm{EBV}}_{{{\mathrm{dams}} }} }}^{2} + \overline{{{\mathrm{PEV}}}} _{{{\mathrm{dams}}}} }}{4} + \bar{\upsigma }_{{{\mathrm{gamMS}}_{{{\mathrm{sires}} }} }}^{2} + \bar{\upsigma }_{{{\mathrm{gamMS}}_{{{\mathrm{dams}} }} }}^{2} \\ \end{aligned} $$

**Explanation of the mate allocation algorithm**

We drew as many pairwise observations from a bivariate normal distribution as number of matings. This was done by drawing random numbers for variable 1 (${A}_{i}$) from a $N\left(\mathrm{0,1}\right)$ distribution. The values for variable 2 (${B}_{i}$) were generated based on the values for variable 1, the desired correlation between the two variables (${r}_{A,B}$) and a $N\left(\mathrm{0,1}\right)$ random independent number (${\beta\:}_{i}$) following Leroy et al. [27]: ${\mathrm{B}}_{{\mathrm{i}}} = {\mathrm{r}}_{{{\mathrm{A}},{\mathrm{B}}}} {\mathrm{A}}_{{\mathrm{i}}} + \sqrt {\left( {1 - {\mathrm{r}}_{{{\mathrm{A}},{\mathrm{B}}}}^{2} } \right)} \beta _{{\mathrm{i}}}$.

The correlation between the two variables of the bivariate distribution was set to the required correlation between EBVs of males and females. Numbers drawn for the first variable were ordered increasingly and numbers of the second variable were reordered according to the reranking done for values of the first variable. The vectors of EBV of males and females were also both sorted increasingly. In the next step, the EBVs of females were reordered according to the order of numbers of the second variable. The order of EBVs in both the vector pertaining to males and females is then the mating plan, i.e., the male at position 1 is mated with the female at position 1 and so forth. An example is given in Table 4. Because the mating strategy involves drawing random numbers, the solution is not unique, i.e., a different solution may be obtained when the procedure is repeated. This was utilized to fine-tune the mating plan by calculating the realized correlation between male and allocated female EBVs and repeating the mate allocation procedure until the realized correlation deviated less than 0.02 from the required correlation. An R script to replicate the described steps is provided in Additional File 7 Code S6.

**Table 4:** Example of the assortative mate allocation strategy

A	EBVs males	Sire1 106	Sire2 103	Sire3 110	Sire3 110	Sire4 108
B	EBVs females	Dam1 95	Dam2 103	Dam3 105	Dam4 98	Dam5 112
C	EBVs males (A) sorted	Sire3 110	Sire3 110	Sire4 108	Sire1 106	Sire2 103
D	EBVs females (B) sorted	Dam5 112	Dam3 105	Dam2 103	Dam4 98	Dam1 95
E	Variable 1	0.1	−0.2	0.5	0.7	0.9
F	Variable 2	−0.3	0	0.4	-0.5	1.8
G	Order variable 1 (E)	2	1	3	4	5
H	Variable 1 (E) ordered according to order of variable 1 (G)	−0.2	0.1	0.5	0.7	0.9
I	Variable 2 (F) ordered according to order of variable 1 (G)	0	−0.3	0.4	−0.5	1.8
J	New order of variable 2 (I)	4	2	1	3	5
K	Sorted EBVs of females (D) ordered according to new order of variable 2 (J)	Dam498	Dam3105	Dam5112	Dam2103	Dam195
		Mating1	Mating2	Mating3	Mating4	Mating5
Sires (C)		Sire3 110	Sire3 110	Sire4 108	Sire1 106	Sire2 103
Dam (K)		Dam4 98	Dam3 105	Dam5 112	Dam2 103	Dam1 95

In this example, 5 matings are to be conducted. 5 dams were selected and 4 sires were selected with Sire3 having a twice as high contribution to the next generation than the other sires. The steps to take are in rows A to K.
