# Supplementary material for: Restructuring breeding programs 2: Assortative mating for improved commercial genetic gain when using optimum contribution selection and diversity introduction
Source: Genet Sel Evol. 2026 May 27;58:32. doi: 10.1186/s12711-026-01049-6 (PMC13263942; doi:10.1186/s12711-026-01049-6)
Supplement: Supplementary file 1 — Additional file 1: Supplementary Fig. S1, S2 and S3. The file also contains detailed description of positive correlation between EBVs of males and females they were mated to under random mating, and explanation of this phenomenon with larger full sib groups (Text S1). The file also contains a detailed description of an idea to combine the mate allocation and selection problem (Text S2). [file 12711_2026_1049_MOESM1_ESM.docx]

|  | Correlation between EBV of sire and dam | Resulting correlation between TBV of sire and dam |
| --- | --- | --- |
| Correlation in matings of current generation | 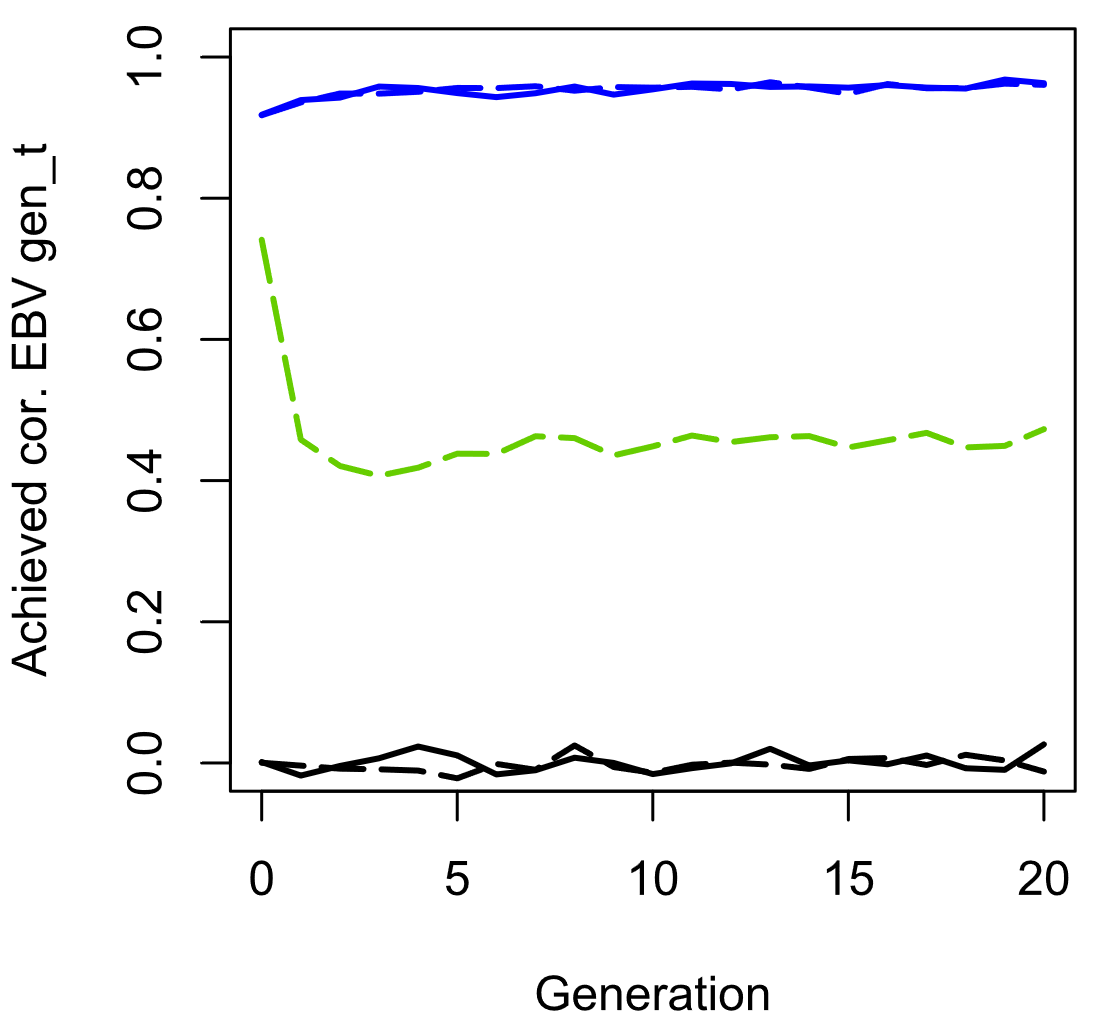  A | 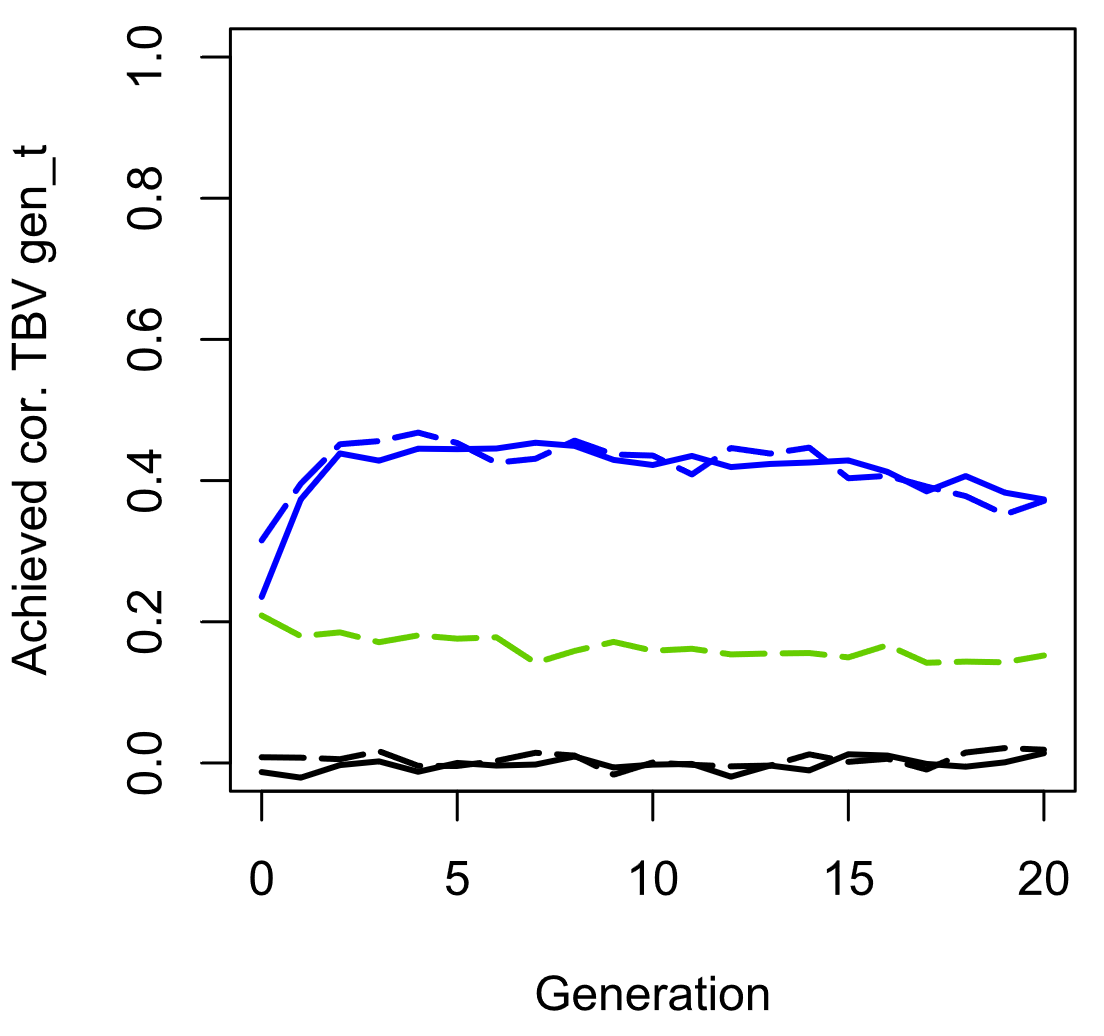  B |
| Correlation in realized matings of last-generation matings | 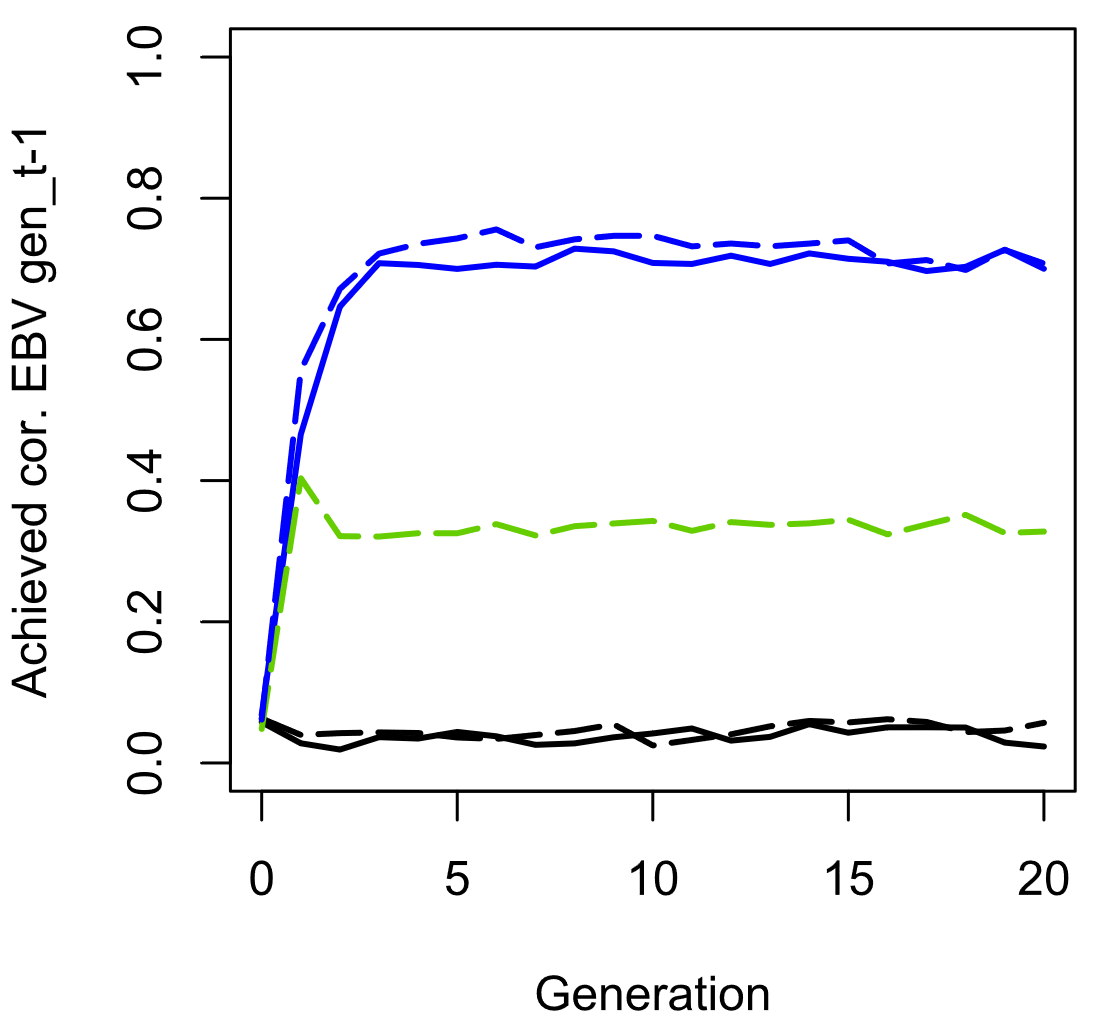  C | 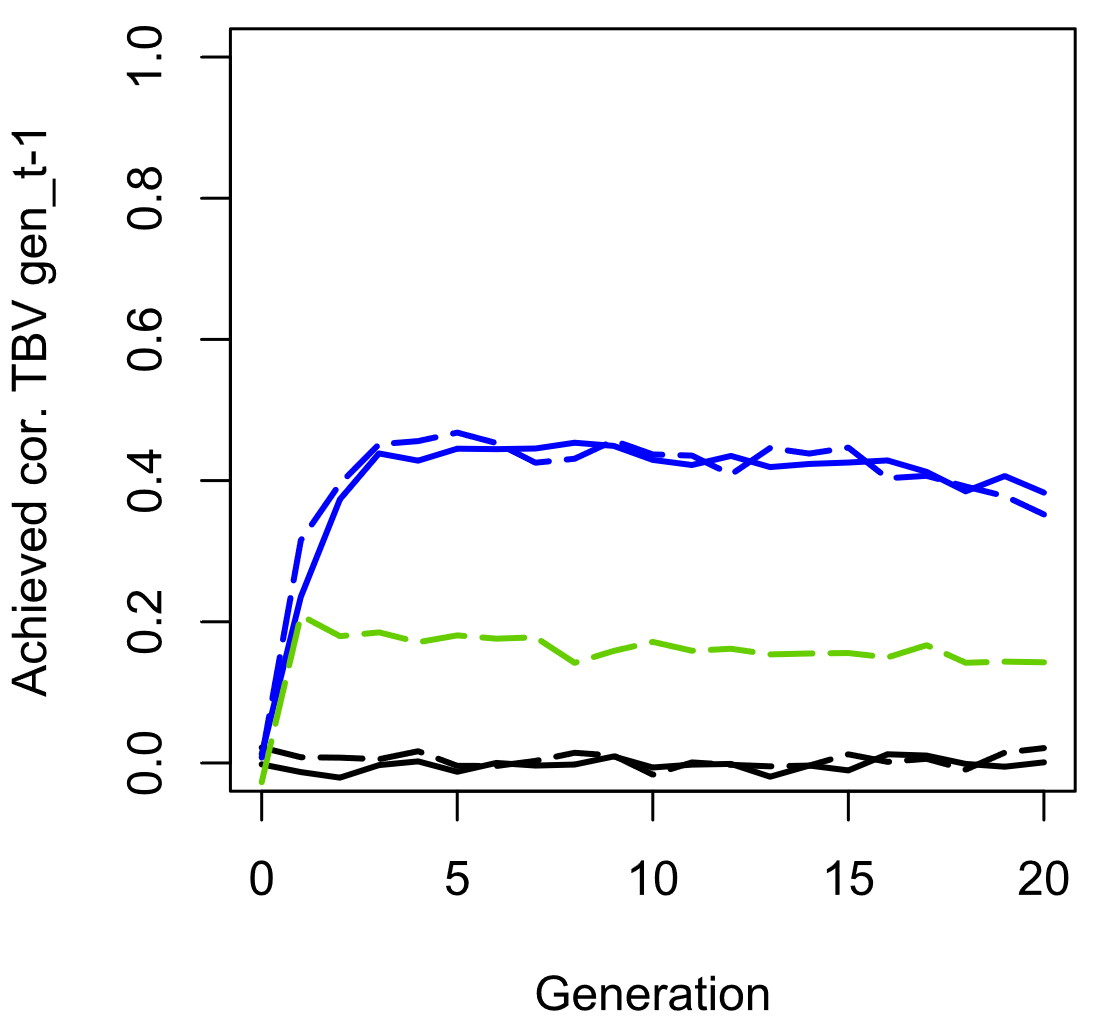  D |
| 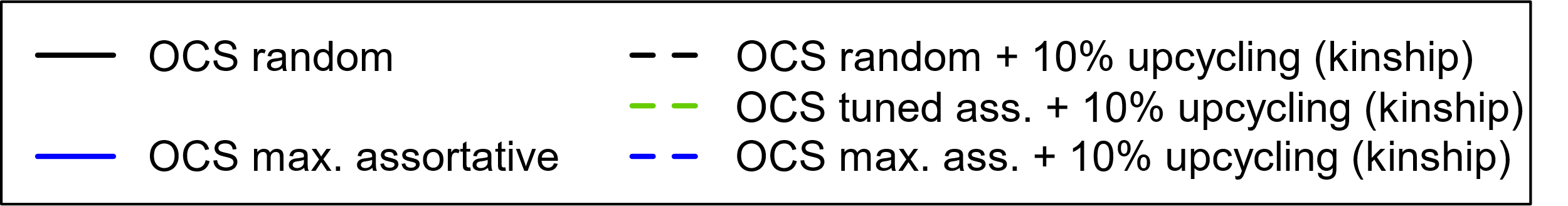 | | |
| Supplementary Figure S1: Correlations between breeding values of males and females they are mated to. Plot A and C show correlations between EBVs and plot B and D show correlations between TBVs. Plot C and D show correlations that are observed in the current generation (t) for matings that were conducted one generation ago (t-1), i.e., after the EBVs of parents were updated with the phenotype information of their offspring. | | |

| 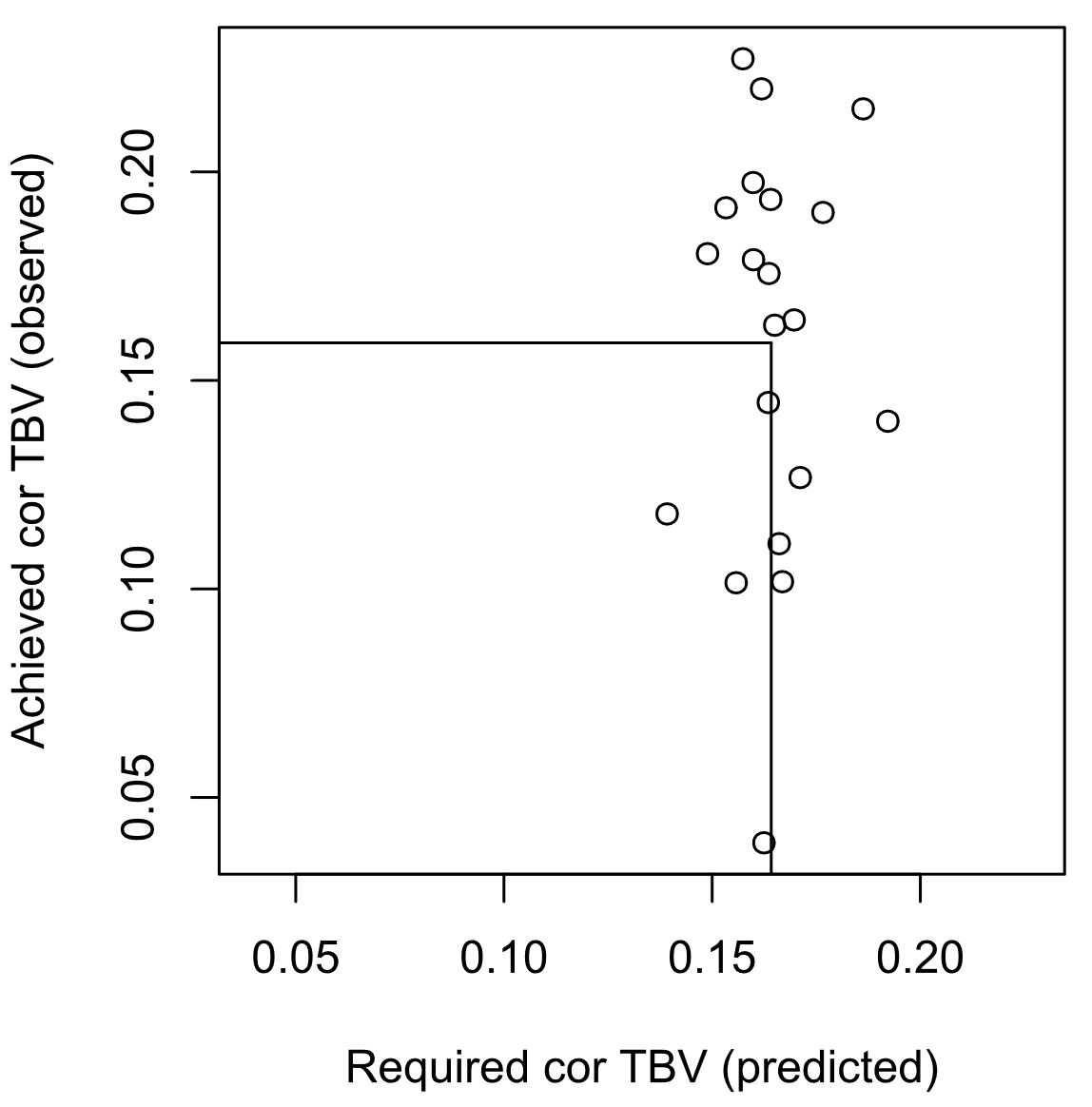 |
| --- |
| Supplementary Figure S2: Required correlation between TBVs of sires and dam versus the achieved correlation between TBVs of sires and dams. Shown are values from the 20 replicates in generation 10. Required correlation is predicted based on the correlation between EBV of sire and dam and the product of the accuracy within the group of selected sires and dams. The achieved correlation is calculated by looking up the sire and dam of all newborn piglets and calculating the correlation between the TBVs of the parents.  Solid lines indicate the average over the replicates. |

| 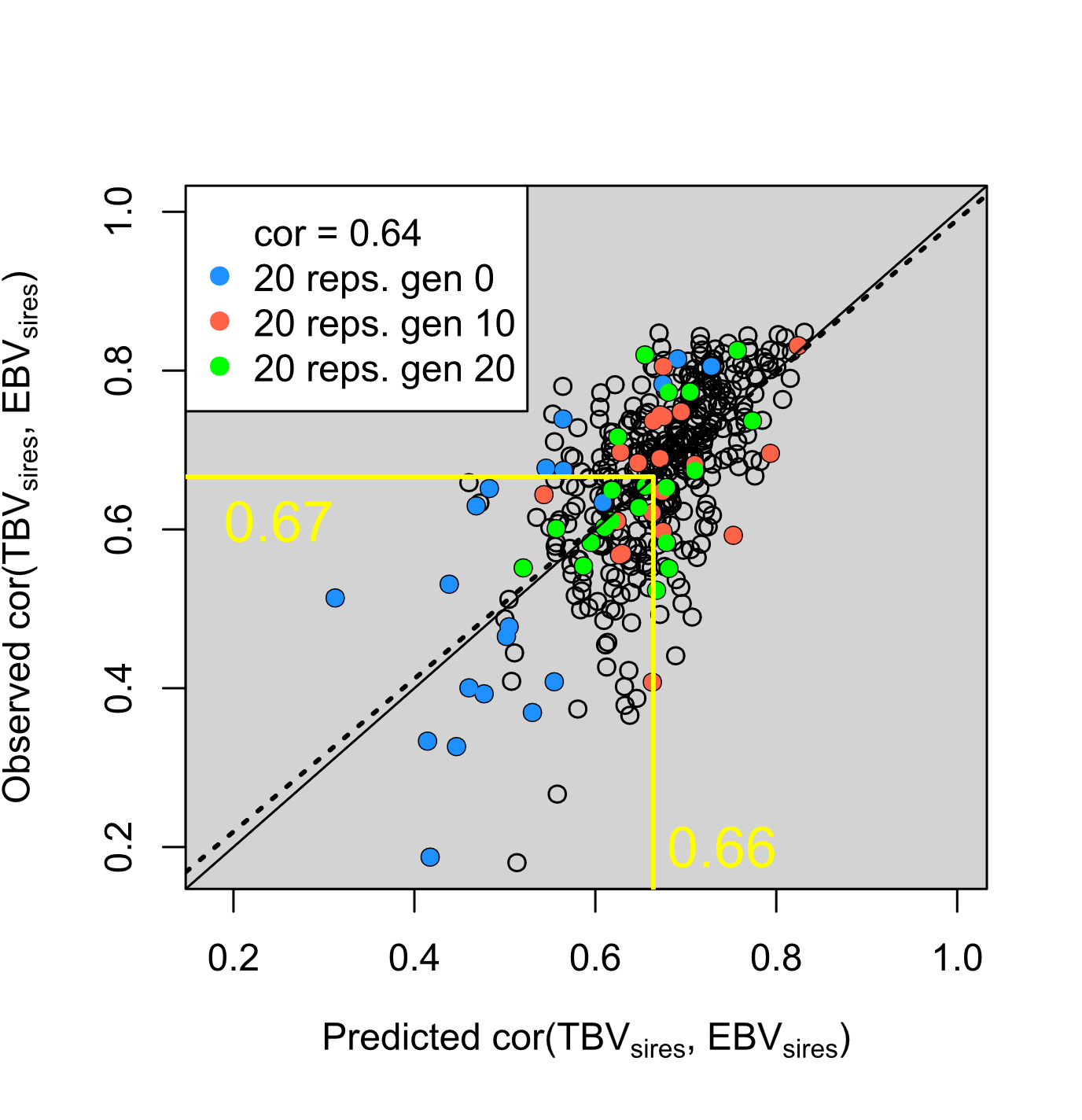  A | 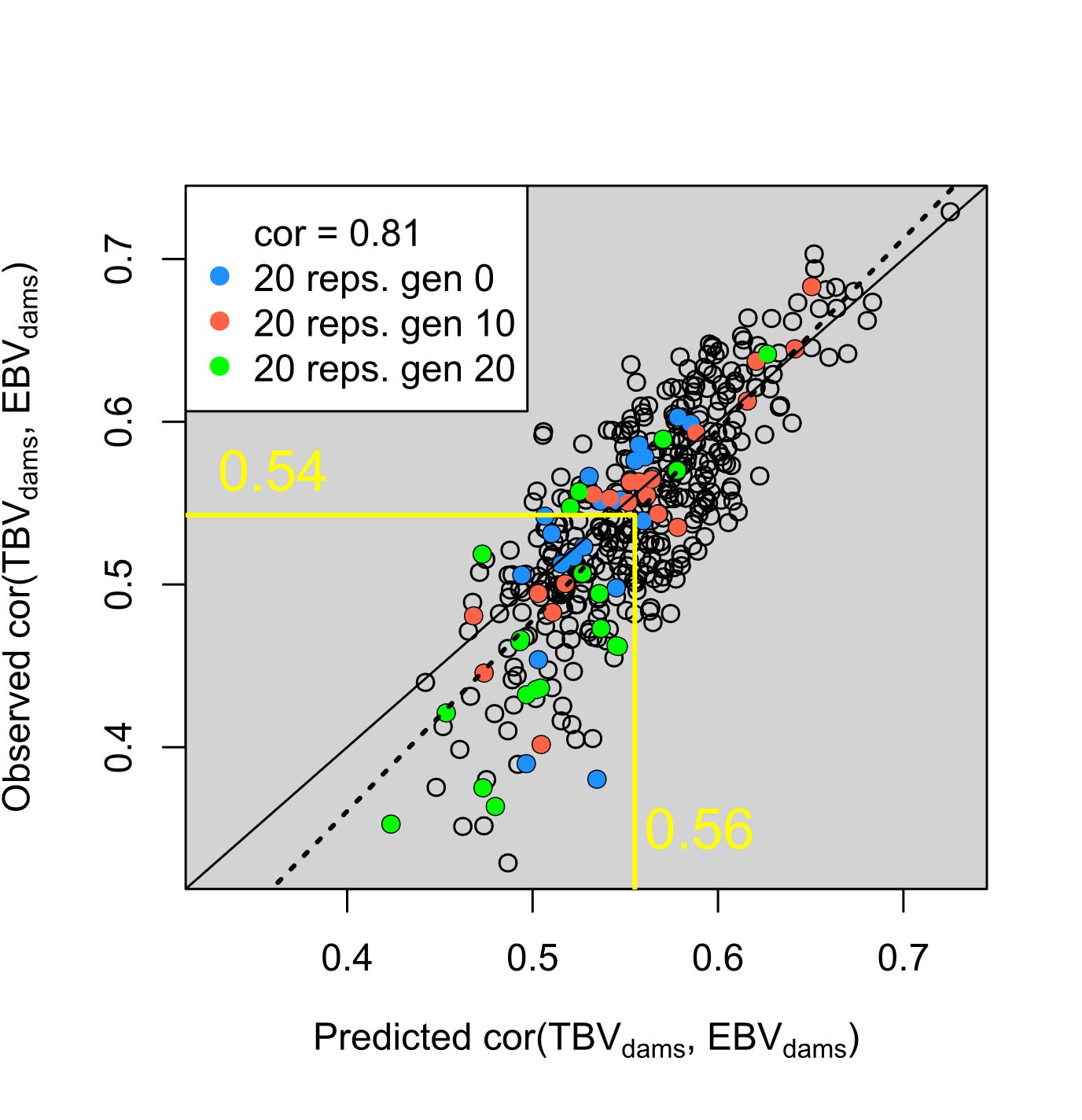  B |
| --- | --- |
| Supplementary Figure S3: Predicted versus observed accuracy of breeding values within the group of selected males (A) and selected females (B) adjusted for varying genetic contributions to the next generation (only males). The predicted accuracies are those used in the denominator in Equation 7. The accuracy was predicted based on the variance of EBV and the PEV obtained from the model-based individual reliability estimate of selected animals. The observed correlation between TBVs and EBVs was calculated as the correlation between TBVs and EBVs of sires (dams) of newborn piglets to obtain the realized accuracy corrected for varying contributions of parents. Every points represents 1 of the 20 replicates in generation 0 – 20 from the tuned assortative mating strategy. Observations from generation 0, 10, and 20 are highlighted. The solid back line is the ideal regression line with intercept of 0 and slope of 1. The dotted line is the regression of observed correlations on predicted correlations. Yellow lines and numbers indicate the average observed and predicted accuracy. | |

**Supplementary Text S1**

**Significant positive correlation of EBVs under random mating with multiple offspring per litter**

We even detected a low but significant positive correlation of EBVs under random mating when looking in the matings of the past generation, which was not observed in the TBVs, and is thus not meaningful (black lines in C and D Supplementary Figure S1). This correlation was approximately 0.03-0.06 and 0.02-0.04 in scenarios with and without diversity introduction, respectively. To clarify, this is the correlation between sire and dam EBVs after the genomic prediction model has been updated with the genotypes and phenotypes of their offspring. Further investigation (not shown) showed that this correlation arises because females are mated to only one male. When increasing the litter size to 30, this correlation could be increased to up to 0.15. However, when all offspring of a female are sired by different males, this correlation is 0. Thus, we conclude that the information of offspring performance back-informs the EBV of their parents. And since all offspring are from the same sire, the sire’s BV influences the EBV of the dam. I.e., if a female is mated to a better than average male by chance, her EBV will be overestimated, such that her EBV will be estimated to be closer the BV of the sire.

**Supplementary Text S2**

**Idea for combination of mate allocation and simultaneous selection**

The mating problem and the selection problem could be solved simultaneously. This could be done by choosing a selection threshold for the AI boars in the next generation followed by calculating the probability of offspring of every possible mating to show a breeding value higher than the threshold as done in [1]. For this, the breeding values and the predicted variance of breeding values of offspring of a mating are needed. These probabilities could be listed in a matrix with the sire of the respective mating in the columns and the dams in the rows. OCS then needs to maximize for the average of this matrix instead of the average of the vector of breeding values (note that the OCS solution would be the same to the traditional method using a vector of EBVs if the matrix listed the parent average breeding values instead of probabilities). The probability of each selected mating then needs to be multiplied with the number of offspring per mating, in this study 6. To get the total number of animals with EBV higher than the threshold, this must be summed over all matings. To get the required number of AI boars to sell semen to production farms of, in this study 80, the threshold used to calculate the probability needs to be adjusted and the previous steps need to be repeated until a solution is found that approximately results in the required number of boars. To our knowledge, only the software mateSel [2, see p. 102 in the software instructions] could solve the described problem and return a mating list. optiSel [3] could maximize for a matrix as described but is not able to optimize mate allocation. In other words, solutions from optiSel would always assume random mating and may thus be worse than mating-specific solutions that could be obtained with mateSel. However, we would expect the benefit from the described more elaborate scheme to be only marginally better. This described strategy may be substitute for our “maximum assortative mating” strategy as it directly aims for the highest genetic level of the best animals in the next generation by choosing animals and matings accordingly. For this reason, this strategy would not be a substitute for our “tuned” approach.

1. Niehoff TAM, ten Napel J, Bijma P, Pook T, Wientjes YCJ, Hegedűs B, et al. Improving selection decisions with mating information by accounting for Mendelian sampling variances looking two generations ahead. Genet Sel Evol. 2024;56:41.

2. Kinghorn BP, Kinghorn AJ. Instructions for Matesel 2024 [Available from: <https://matesel.com/content/documentation/MateSelInstructions.pdf>. Accessed Sep 03, 2024]

3. Wellmann R. Optimum contribution selection for animal breeding and conservation: the R package optiSel. BMC Bioinformatics. 2019;20:25.
